# Metabolic Engineering of *Nicotiana benthamiana* to Produce Cannabinoid Precursors and Their Analogues

**DOI:** 10.3390/metabo12121181

**Published:** 2022-11-25

**Authors:** Vaishnavi Amarr Reddy, Sing Hui Leong, In-Cheol Jang, Sarojam Rajani

**Affiliations:** 1 Temasek Life Sciences Laboratory, 1 Research Link, National University of Singapore, Singapore 117604, Singapore; 2 Department of Biological Sciences, National University of Singapore, Singapore 117543, Singapore

**Keywords:** cannabis, cannabinoids, *N. benthamiana*, *N. benthamiana* cell cultures, metabolic engineering

## Abstract

In recent years, the perspective towards the use of cannabis has slowly shifted from being an illicit drug to a medicinal plant. The pathway and enzymes involved in the production of cannabinoids are known; however, studies evaluating the production of cannabinoids in heterologous plants and cell cultures are still limited. In this study, we assessed the potential use of *N. benthamiana* (*Nicotiana benthamiana*) plants as a heterologous host for producing natural and novel cannabinoids. Transgenic *N. benthamiana* plants expressing genes encoding cannabis acyl-activating enzyme and olivetol synthase were generated, which were then used for transiently expressing other downstream pathway genes. Production of olivetolic acid and divarinic acid, the universal precursors for major and minor cannabinoids, respectively, was observed in transgenic *N. benthamiana* plants. To produce novel cannabinoid precursors with different side chains, various fatty acids were infiltrated into the transgenic *N. benthamiana* plants and the production of novel derivatives was observed. Although we were not able to derive the core intermediate, cannabigerolic acid, from our transgenic plants, possibly due to the low production levels of the precursors, our transgenics plants still serve as a high-potential platform for further development and exploring the *N. benthamiana* chemical space for generating novel cannabinoids.

## 1. Introduction

Cannabis plants produce a unique class of compounds known as phytocannabinoids that possess medicinal and psychotropic properties. More than 100 different cannabinoids have been reported, of which the most prominent ones are delta-9-tetrahydrocannabinol (THC) and cannabidiol (CBD). THC is the compound responsible for the psychotropic and toxic effects of cannabis. However, the presence of non-intoxicating compounds such as CBD, cannabichromene (CBC), cannabigerol (CBG) and others have fueled research on the use of cannabinoids for medicinal purposes. The potential application of cannabis-based medicine for the treatment of chronic diseases such as Parkinson’s disease, epilepsy, cancer, pain management and brain injury has given momentum to further understand the pathways producing the different cannabinoids and their pharmacological properties [1,2,3,4,5,6,7].

**Figure 1:**
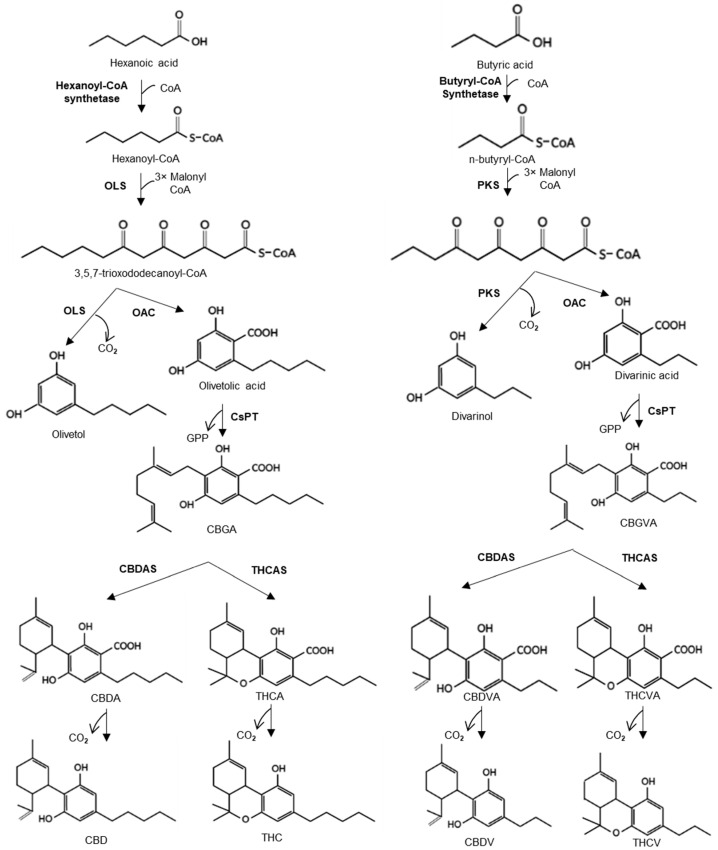
Cannabinoid pathway. Pathway showing the production of cannabinoids via the polyketide and MEP pathway. In the polyketide pathway, acyl-activating enzyme (AAE), a hexanoyl-CoA synthetase, produces hexanoyl-CoA from hexanoic acid via the fatty acid synthesis pathway. Subsequently, 3,5,7-trioxododecaneoyl-CoA is synthesised by olivetol synthase (OLS) through the sequential condensation of hexanoyl-CoA with three molecules of malonyl-CoA. Thereafter, olivetolic acid cyclase (OAC) catalyses the cyclization to olivetolic acid (OLA). Following this, OLA is prenylated by geranyldiphosphate:olivetolate geranyltransferase (CsPT) using geranyl diphosphate (GPP) derived from the MEP pathway. Cannabigerolic acid (CBGA) formed from this reaction then serves as an intermediate for the formation of cannabinoids with pentyl side chains. Two major cannabinoids formed are the tetrahydrocannabinolic acid (THCA) and cannabidiolic acid (CBDA) which become tetrahydrocannabinol (THC) and cannabidiol (CBD), respectively through the process of decarboxylation. Cannabionoids with propyl side chains are derived from the precursor divarinic acid (DA), which is derived from the conversion of butyric acid to n-butyryl-CoA via a butyryl-CoA synthetase. Thereafter, a type III polyketide synthase (PKS) and OAC form DA. Subsequently, it can lead to the formation of tetrahydrocannabivarinic acid (THCVA) and cannabidivarinic acid (CBDVA), which are decarboxylated to tetrahydrocannabivarin (THCV) and cannabidivarin (CBDV), respectively.

Cannabinoids are structurally isoprenylated resorcinyl polyketides that are biosynthesised in the glandular trichomes of female flowers [8,9] and have been classified into nine structural families: namely, THC, CBD, cannabichromene (CBC), cannabinodiol (CBND), cannabigerol (CBG), cannabitriol (CBT), cannabicyclol (CBL), cannabielsoin (CBE) and cannabinol (CBN) [10]. All cannabinoids share the same initial pathways, namely the polyketide and methylerythritol 4-phosphate (MEP) pathway, which produce olivetolic acid (OLA) and geranyl diphosphate (GPP), respectively. The polyketide pathway begins with the formation of hexanoic acid through the fatty acid biosynthesis pathway, which is then converted to hexanoyl-CoA by an acyl-activating enzyme (AAE) [11] (Figure 1). Thereafter, a type III polyketide synthase (PKS) named olivetol synthase (OLS) catalyses the sequential condensation of hexanoyl-CoA with three molecules of malonyl-CoA to yield olivetol [12]. This is then cyclised to olivetolic acid (OLA) by olivetolic acid cyclase (OAC) [13]. A geranyldiphosphate:olivetolate geranyltransferase (CsPT) then prenylates OLA using GPP derived from the plastidial MEP pathway to generate cannabigerolic acid (CBGA), the common precursor of cannabinoids [14,15,16,17].

The cannabinoid synthase enzymes tetrahydrocannabinolic acid (THCA) synthase, cannabidiolic acid (CBDA) synthase, and cannabichromenic acid (CBCA) synthase act on CBGA to produce THCA, CBDA and CBCA, respectively. These common cannabinoids are known as C5 phytocannabinoids, as their resorcinyl alkyl side-chain has a pentyl (C_5_) configuration. C3 phytocannabinoids with propyl (C_3_) configuration have also been reported as minor cannabinoids in a few germplasms [18,19]. Divarinol, which is the precursor of cannabinoids with propyl side chains, is produced from n-butyryl-CoA, which is catalysed by AAE, also known as butyryl-CoA synthetase from butyric acid. Later, a type III PKS, along with OAC, forms divarinic acid (DA). OLS has been shown to accept n-butyryl-CoA and produce divarinol and DA [12,16]. DA is then prenylated with GPP to form cannabigerovarinic acid (CBGVA), the precursor for C3 phytocannabinoids. The cannabinoid synthase enzymes can convert CBGVA into the propyl homologues of THCA, CBDA, and CBCA, known as tetrahydrocannabivarinic acid (Δ^9^-THCVA), cannabidivarinic acid (CBDVA), and cannabichromevarinic acid (CBCVA), respectively [20]. Apart from C5 and C3, a variety of odd- and even-carbon-length cannabinoids have been reported as minor constituents in a few germplasms; however, information about their substrates and catalytic enzymes is limited [21,22]. The length of the cannabinoid alkyl side-chain is known to influence receptor binding affinity, hence its biological/pharmacological activity [23].

The cannabinoids that have been studied the most for medicinal applications are THC and CBD. Studies on the pharmacological effects of minor cannabinoids are greatly impaired due to their low abundance [24]. As the use and acceptance of medicinal cannabis grow, it will be important to analyse all the different cannabinoids produced to fully exploit the therapeutic potential of the cannabis plant. Metabolic engineering and synthetic-biology-based approaches provide an opportunity to produce pure and high quantities of individual cannabinoids, especially those that are rare in the plant. A recent review highlights the heterologous approaches carried out to date for the production of cannabinoids [25]. Metabolic engineering of plants and plant suspension cultures for the production of natural products is widely known and serves as a promising heterologous system to produce high-value metabolites [26]. Among model plants, *N. benthamiana* is commonly used for the heterologous production of metabolites. 

Few studies have investigated the expression of cannabinoid pathway enzymes in *N. benthamiana*. *N. benthamiana* was first used to express CBDA synthase [27]. Later, THCA synthase was successfully expressed in *N. benthamiana* hairy root cultures and in *N. benthamiana* plants [28,29,30]. Recently, metabolic engineering of the cannabinoid pathway was attempted in *N. benthamiana* by transiently expressing the *AAE1*, *OLS* and *OAC* genes along with infiltration of hexanoic acid which led to the production of mainly OA-glucoside. Interestingly, when *CsPT4* and *THCAS* were also expressed with the above genes, CBGA or THCA formation was not observed. The lack of CBGA/THCA production was attributed to the glucosylation of OA, which hinders the downstream biosynthesis of cannabinoids. The intrinsic glucosylation of various cannabinoid pathway intermediates was also determined by leaf infiltration of OA, CBGA and THCA individually. The formation of OA-glucosides and CBGA-glucosides was observed but THCA-glucosides were not seen [17].

In this study, we engineered *N. benthamiana* to stably express the cannabis genes *AAE* and *OLS* to produce olivetol with the infiltration of hexanoic acid. The stable transgenics were then used to transiently express *OAC*, which led to the production of OA in *N. benthamiana* leaves. Towards the production of minor cannabinoids, butanoic acid was infiltrated and the production of DA was observed. Further, different fatty acids were infiltrated into *N. benthamiana* leaves, which led to the production of various novel cannabinoid precursors. From these stable transgenics, *N. benthamiana* cell lines were also established and evaluated for their ability to produce olivetol. Analysis showed that the amount of olivetol produced by transgenic *N. benthamiana* plants appeared higher than the cell lines, but further comparative analysis needs to be performed. Our results show the feasibility of exploring *N. benthamiana’s* chemical space for creating novel cannabinoids and for elucidating the structural aspects of the substrate selectivity of the OLS enzyme.

## 2. Materials and Methods

### 2.1. Plant Material and Transformation

*N. benthamiana* transformation was performed as described previously with minor modifications [31]. Briefly, leaves from three-week-old *Nicotiana benthamiana* grown in tissue culture conditions were used as explants for transformation. The explants were dipped in diluted *Agrobacterium* cells in Murashige and Skoog (MS) media for 10 min before transferring to MS plates containing 6-benzylaminopurine (BA, 1 mg/L) and 1-naphthaleneacetic acid (NAA, 0.1 mg/L) for 2 days. The explants were washed several times with water, followed by MS media with cefotaxime (Ctx), and allowed to incubate for 30 min. Explants were then transferred to MS plates with BA (1 mg/L), NAA (0.1 mg/L), Ctx (250 mg/L), and kanamycin (20 mg/L). After 2 to 3 weeks, explants were transferred to shoot regeneration media [BA (1 mg/L), NAA (0.1 mg/L), Ctx (125 mg/L), kanamycin (20 mg/L)]. Once shoots were formed, they were transferred to rooting media [NAA (0.1 mg/L), Ctx (125 mg/L), kanamycin (20 mg/L)]. Positive plants were visually selected using a GFP filter. 

### 2.2. Generation of Transgenic N. benthamiana Calli and Cell Suspension Cultures

Calli were generated from leaves of transgenic and wild-type (WT) *N. benthamiana* using an approach specified previously [32]. Briefly, seeds were sterilised and germinated for eight weeks before the leaves were cut into smaller pieces and placed onto callus generation plates. The media components used were the same as previously described [32] with the addition of 20 mg/L kanamycin and 125 mg/L Ctx for the transgenic lines. After three to four weeks, when the calli began to form, the explants were then transferred to new callus generation plates with reduced 2,4-dichlorophenoxyacetic acid (0.2 mg/L). These plates were labelled as maintenance plates. Once the calli were well developed, appearing as a white or light yellow in colour and with soft texture, the calli formed from transgenic lines were visually screened for GFP expression. Calli with the highest intensity of GFP expression were picked and propagated monthly on maintenance plates. 

Subsequently, calli were taken from the plates and grown in flasks to generate cell suspensions. The suspensions were labelled as WT, line A, line B, line C and line D. The media components used were the same as previously described [32] with the addition of 20 mg/L kanamycin and 125 mg/L Ctx for the transgenic cell lines. For the generation of cell cultures, the calli were broken down by resuspending through pipetting. Cells were grown at 28 °C with shaking at 140 rpm. They were subcultured every one to two weeks depending on the density of the cultures. 

### 2.3. Vector Construction

Sequences of *OLS*, *AAE*, *OAC*, *CsPT1* and *CsPT4* were obtained from the genome of *Cannabis sativa* deposited in NCBI, and the genes were synthesised from GenScript, Singapore. Accession numbers of the gene sequences used are provided under the data availability statement. Full-length open reading frames (ORFs) of the genes were amplified using Phusion high-fidelity polymerase (Thermo Fisher Scientific, Waltham, MA, USA). OLS, AAE, TNos terminator and *Arabidopsis* ribulose bisphosphate carboxylase small chain 2B (AtRBCS2B) promoter [33] were then cloned into a modified pENTR vector containing multiple cloning sites (MCS) using restriction enzyme digestion and ligation. Restriction enzymes AvrII and SphI, BamHI and BglII, XhoI and SbfI, SalI and NdeI were used to clone OLS, TNos terminator, AtRBCS2B promoter and AAE, respectively. Plasmids from the positive clones were isolated and cloned into the destination vector PK7WG2D using LR recombination. The destination vector containing *OLS* between the CaMV 35S promoter and TNos terminator, and *AAE* between AtRBCS2B promoter and 35S terminator, was labelled as PK7VRA (Figure S1a). Full-length ORF of *OAC*, *CsPT1* and *CsPT4* was cloned into pENTR vector using the *pENTR*™/D-TOPO^®^ Cloning Kit (Thermo Fisher Scientific, Waltham, MA, USA) and later transferred into PBADC vector. All the destination vectors were verified by DNA sequencing and transformed into *Agrobacterium* EHA105 strain by a heat shock method. The transformed EHA105 cells were used to generate stable transgenic *N. benthamiana* plants expressing *OLS* and *AAE*. Primers used in this study are listed in Table S1. The complete sequence of the vector PK7VRA is shown in File S1.

### 2.4. Southern Blot Analysis

Genomic DNA (gDNA) was extracted from transgenic *N. benthamiana* lines and WT plants using the cetyl trimethylammonium bromide method. A total of 10 µg of gDNA samples were digested with Sal I enzyme and resolved on 0.8% agarose gel alongside digoxigenin (DIG)-labelled DNA molecular weight marker II (Roche, Mannheim, Germany). The agarose gel was treated, firstly by depurination with 0.2 M Hydrochloric acid (HCl), followed by denaturation (0.5 M Sodium hydroxide (NaOH); 1.5 M HCl). Lastly, the gel was neutralised [1 M (hydroxymethyl) aminomethane hydrochloride (Tris-HCl), pH 7.4; 1.5 M Sodium Chloride (NaCl)] before transferring to a positively charged nylon membrane, Hybond^®^ N+ (Whatman^®^, Maidstone, UK). Hybridization was performed using a DIG-labelled P35S-probe-targeting CaMV 35S promoter which was prepared using a DIG-labelling kit (Roche, Switzerland). Finally, chemiluminescence on the membrane was detected using ChemiDoc Touch Imaging System (Bio-Rad, Hercules, CA, USA).

### 2.5. In Vivo Assay and Liquid Chromatography–Mass Spectrometry (LC-MS) Analysis of Transgenic N. benthamiana Leaves

For in vivo assay, *Agrobacterium* cultures harbouring plasmids *35S_pro_*:*OAC*, *35S_pro_*:*CsPT1*, *35S_pro_*:*CsPT4* and silencing suppressor *35S_pro_*:*p19* were pelleted and resuspended in MMA (10 mM MES, 10 mM MgCl_2_, 100 µM acetosyringone) solution to OD_600_ = 1. The solutions were then incubated at room temperature for 3 h and later mixed at a 1:1 or 1:1:1 ratio and infiltrated into transgenic *N. benthamiana* leaves using a 1 mL needleless syringe. After 2 d, 1 mM of fatty acid substrates dissolved in 5% ethanol were infiltrated into the leaves using a 1 mL needleless syringe. After 24 h, 400 mg of co-infiltrated leaves were macerated with 20% methanol and incubated for 1 h at room temperature on a shaking platform. The homogenate was then centrifuged at 14,000× *g* rpm for 10 min and the supernatant was evaporated using a vacuum evaporator. The dried sample was dissolved in 200 µL of methanol, which was passed through a 0.22 µm cellulose acetate Costar Spin-X centrifuge filter (Corning, Glendale, AZ, USA) before loading 5 µL into LC-MS system. LC-HRMS and Q Exactive Plus mass spectrometer (Thermo Fisher Scientific, Waltham, MA, USA) were used to analyse the samples. Separation was achieved using an Accucore RP-MS column (2.6 μm, 2.1 × 100 mm) (Thermo Fisher Scientific, Waltham, MA, USA) and mobile phases consisted of 0.05% HCOOH in water (A) and 0.005% HCOOH in Acetonitrile (B). Gradient conditions were as follows: −3.0–0.0 min equilibration; 0.0–9.0 min 5–95% B, 9.0–10.0 min 95–5% B. The flow rate of the mobile phase was 0.3 mL/min and the column temperature was maintained at 27 °C. Xcalibur and Freestyle software were used for program setup and data visualization, respectively. Parallel reaction monitoring (PRM) was used to detect the peaks. Details of PRM are shown in Table S2. The compounds were identified by comparison with retention time (RT) and mass spectrum of standards. For peaks where standards were not available, the peaks were confirmed by the presence of signature ion peaks in the mass spectrum which is specific to each peak. 

### 2.6. LC-MS of N. benthamiana Cell Lines

Freshly inoculated cell cultures were grown for 1–2 weeks in the dark. The cultures were then exposed to light for three days, following which 1 mM of hexanoic acid was added to the cultures. After 24 h, 80 mL of each cell culture was divided into two 50 mL falcon tubes. A total of 5 mL of ethyl acetate was added and vortexed vigorously. The mixture was shaken at room temperature for 2 h. The tubes were then centrifuged at 4000× *g* rpm for 15 min. The upper layer (~2 mL) was transferred to a fresh tube and was evaporated using a nitrogen gas evaporator. The dried sample was dissolved in 50 µL of methanol, which was passed through a 0.22 µm cellulose acetate Costar Spin-X centrifuge filter (Corning, Glendale, AZ, USA) before loading 5 µL into the LC-MS system. LC-MS analysis was performed as described previously in Section 2.5.

### 2.7. RNA Isolation, Semi-Quantitative PCR and Quantitative Real-Time PCR (qRT-PCR)

Total RNA was isolated from transgenic leaves or calluses using the RNeasy^®^ Plus Mini kit (Qiagen, Hilden, Germany). An iScript^TM^ cDNA Synthesis kit (Bio-Rad, Hercules, CA, USA) was used to reverse-transcribe 500 ng of RNA to cDNA. Expression levels of genes were analysed using semi-quantitative PCR and qRT-PCR. Semi-quantitative PCR was performed using DreamTaq™ Green Buffer (10X) (Thermo Fisher Scientific, Waltham, MA, USA), dNTP mix (Thermo Fisher Scientific, Waltham, MA, USA), and TLL Taq Polymerase (TLL 047). The cycling profile was an initial denaturation at 94 °C for 5 min, followed by denaturation at 94 °C for 30 s. Annealing of primers occurred at the appropriate primers’ melting temperature for 30 s and extension at 72 °C for 30 s, followed by a final extension time of 10 min at 72 °C. Steps from denaturation to extension were repeated with a varying number of cycles to prevent band saturation. qRT-PCR was performed in a 384-well PCR plate using KAPA SYBR fast master mix (KAPA Biosystems, Wilmington, MA, USA) and ABI PRISM 7900HT real-time PCR system (Applied Biosystems, Waltham, MA, USA). For a total PCR reaction of 5 µL, 0.3 µL of cDNA was used and the cycling profile was set at 50 °C for 2 min, 95 °C for 10 min, 40 cycles of 95 °C for 15 s and 60 °C for 60 s. After the thermal cycles, the dissociation analysis (melting curve) was carried out to confirm the specific amplification of the PCR reaction by adding a profile of 95 °C for 15 s, 60 °C for 15 s followed by a temperature ramp to 95 °C and hold for 15 s. In the current study, *Actin* was used as an internal reference. A non-template reference was included for each gene to eliminate the possibility of contamination of reaction components and primer dimer formation. SDS 2.4 software (Applied Biosystems, Waltham, MA, USA) was used to analyse the obtained results. The threshold cycle (C_t_) value of a gene is the cycle number required for the SYBR Green fluorescence signal to reach the threshold level during the exponential phase for detecting the amount of accumulated nucleic acid [34]. Comparative delta Ct values of target genes to *Actin* were taken as relative expression among different tissues. The amount of target gene, normalised to *Actin* gene, was calculated by 2^-(Ct[target gene]-Ct[*Actin*])^. Error bars represent mean ± SD. All primers used in this study are listed in Table S1.

### 2.8. Statistical Analysis

Data are indicated as “mean ± SD” of three to six biological replicates, each performed in triplicates. 

## 3. Results

### 3.1. Engineering the Cannabinoid Precursor Pathway to Produce OA and DA in Transgenic N. benthamiana Plants

**Figure 2:**
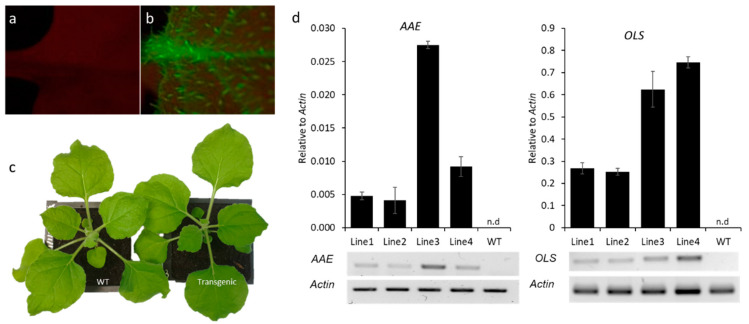
Visual screening and expression analysis of *AAE* and *OLS* in transgenic *N. benthamiana* plants. (**a**,**b**) Representative photo showing the non-fluorescing WT and GFP-fluorescing transgenic *N. benthamiana* leaves. (**c**) Representative photo of WT and transgenic *N. benthamiana* plant. (**d**) qRT-PCR analysis for the expression of *AAE* and *OLS* in the transgenic (Lines 1–4) and WT *N. benthamiana* plants. *Actin* was used as the internal reference. *AAE*; *acyl-activating enzyme*, *OLS*; *olivetol synthase*, n.d; not detected.

Transgenic *N. benthamiana* plants co-expressing *OLS* (1158 bp) and *AAE* cDNAs (2163 bp) under CaMV 35S and AtRBCS2B promoters, respectively, were generated and labelled as PK7VRA plants. Transgenics were selected in kanamycin media and screened visually using a GFP filter (Figure 2a,b). Four lines showing strong GFP fluorescence were selected and labelled as lines 1–4. The number of gene insertions was confirmed by Southern blotting (Figure S1b). Lines 1, 3 and 4 had single insertions each and line 2 had multiple insertions. Transgenics looked phenotypically similar to WT plants (Figure 2c). The ectopic expression of *OLS* and *AAE* genes in transgenics was confirmed by semi-quantitative PCR and qRT-PCR (Figure 2d and Figure S2a). 

**Figure 3:**
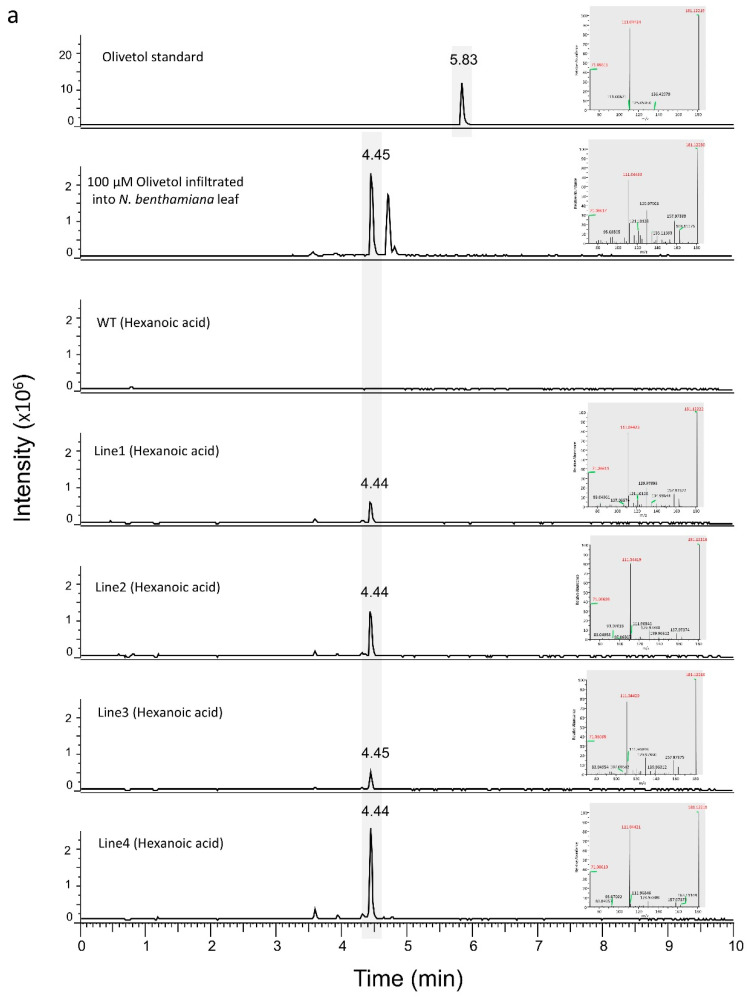

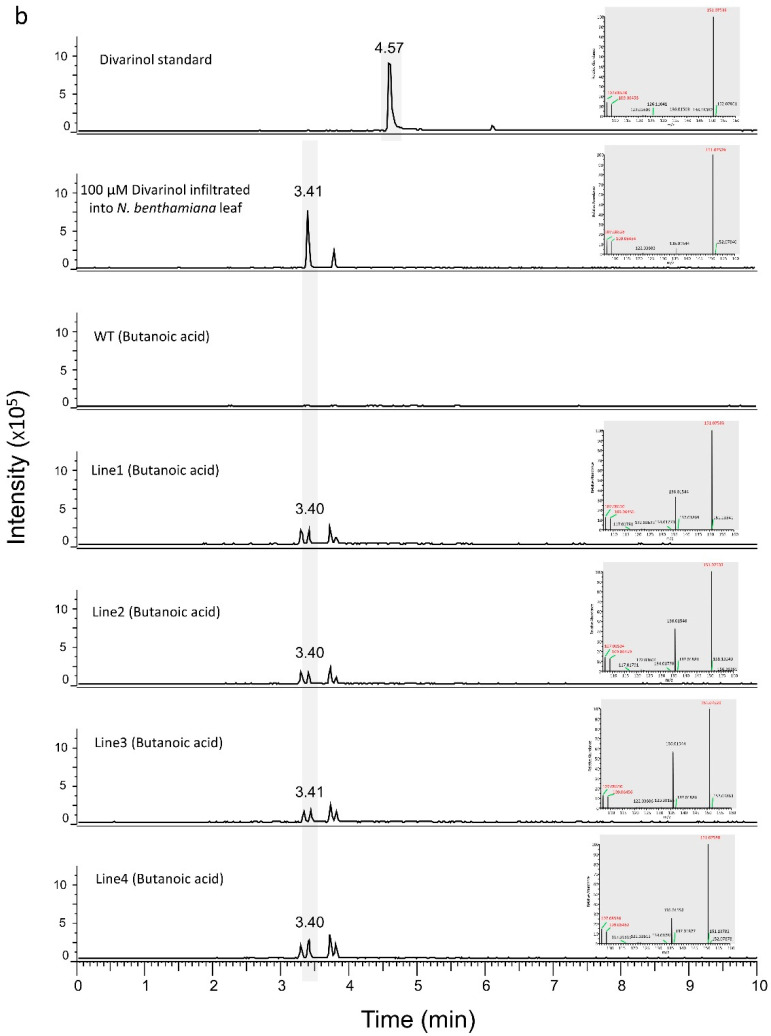
LC-MS results for transgenic and WT *N. benthamiana* leaves infiltrated with hexanoic acid/butanoic acid. (**a**) A total of 1 mM hexanoic acid substrate was infiltrated into the WT and transgenic *N. benthamiana* leaves. The peak of olivetol from the transgenic *N. benthamiana* plants had a forward shift of RT in comparison to olivetol standard. However, when compared with the peak from *N. benthamiana* WT leaves infiltrated with olivetol, a similar shift in RT was observed. (**b**) A total of 1 mM butanoic acid substrate was infiltrated into the WT and transgenic *N. benthamiana* leaves. The peak of divarinol from the transgenic *N. benthamiana* plants had a forward shift of RT in comparison to divarinol standard. However, when compared with the peak from *N. benthamiana* WT leaves infiltrated with divarinol, a similar shift in RT was observed. The signature ions of the representative peaks are shown in red at the right of each chromatogram.

To test for the production of olivetol and divarinol, 1 mM of hexanoic acid or butyric acid was infiltrated into the leaves of transgenics. After 24 h, the leaves were processed and subjected to LC-MS analysis. As shown in Figure 3a,b, leaves of all four transgenic lines infiltrated with hexanoic acid and butyric acid could produce varying amounts of olivetol and divarinol, respectively. The ability of OLS to accept both hexanoic acid and butyric acid as substrates has been previously reported [12]. Peaks of olivetol and divarinol were seen at an earlier RT in the transgenics than that of their respective standards. This might be due to the complex background matrix of the plant sample. Matrix effects are known to alter the RT [35]. To test this, we infiltrated 100 µM of olivetol standard into *N. benthamiana* plants and subjected it to LC-MS after 24 hrs. As seen in Figure 3a,b the peaks of olivetol/divarinol were seen at an earlier RT, which matches with the peaks produced by the transgenic lines. 

**Figure 4:**
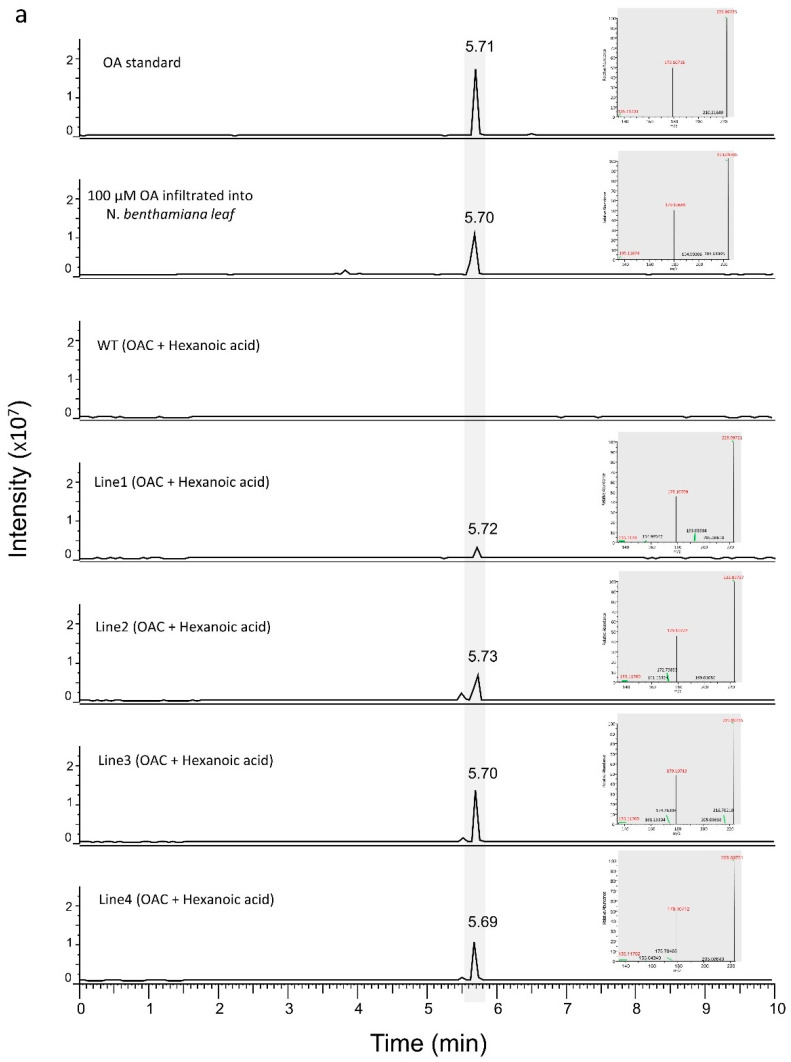

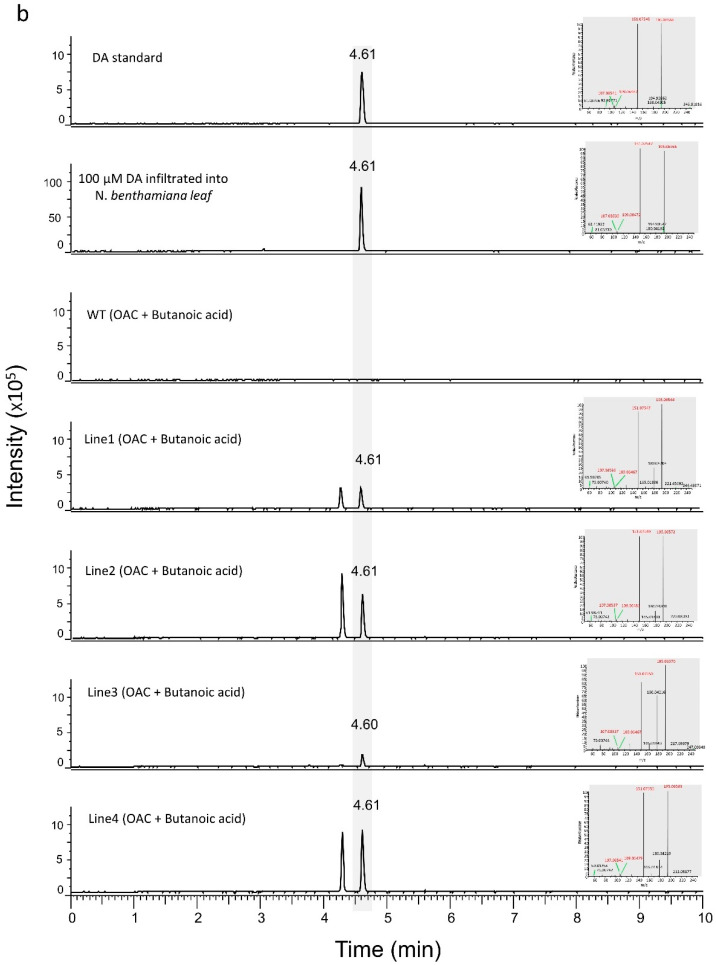
LC-MS results for transgenic and WT *N. benthamiana* leaves infiltrated with *OAC* cultures and hexanoic acid/butanoic acid. (**a**) A total of 1 mM hexanoic acid substrate, along with *OAC*-expressing *Agrobacterium* cultures, were infiltrated into the WT and transgenic *N. benthamiana* leaves. OA was produced in transgenic *N. benthamiana* plants with the same RT as the OA standard. (**b**) A total of 1 mM butanoic acid substrate, along with *OAC*-expressing *Agrobacterium* cultures, were infiltrated into the WT and transgenic *N. benthamiana* leaves. DA was produced in the transgenic *N. benthamiana* plants with the same RT as the DA standard. The signature ions of the representative peaks are shown in red at the right of each chromatogram. *OAC*; *olivetolic acid cyclase*, OA; olivetolic acid, DA; divarinic acid.

To test if we could produce OA and DA, we transiently expressed the *OAC* gene in transgenic *N. benthamiana* plants expressing *OLS* and *AAE*. After two days, the leaves were infiltrated with 1 mM of hexanoic acid or butyric acid and processed for LC-MS analysis after a further 24 h incubation. As seen in Figure 4a,b, transgenic leaves infiltrated with substrates and *OAC* were able to produce OA and DA with hexanoic acid and butyric acid, respectively. The amount of OA produced was calculated for the transgenic *N. benthamiana* plant lines 1, 2, 3 and 4 to be 3.98 ng/g fresh weight (FW), 8.78 ng/g FW, 12 ng/g FW and 7.83 ng/g FW, respectively. The amount of DA produced was calculated for transgenic *N. benthamiana* plant lines 1, 2, 3 and 4 to be 1.12 ng/g FW, 3.06 ng/g FW, 0.65 ng/g FW and 4.41 ng/g FW, respectively. Peaks were confirmed by RT of standards and by the presence of signature ions in the mass spectrum which were specific to each metabolite.

### 3.2. Production of Analogues of Cannabinoid Precursors in Transgenic N. benthamiana Plants

**Figure 5:**
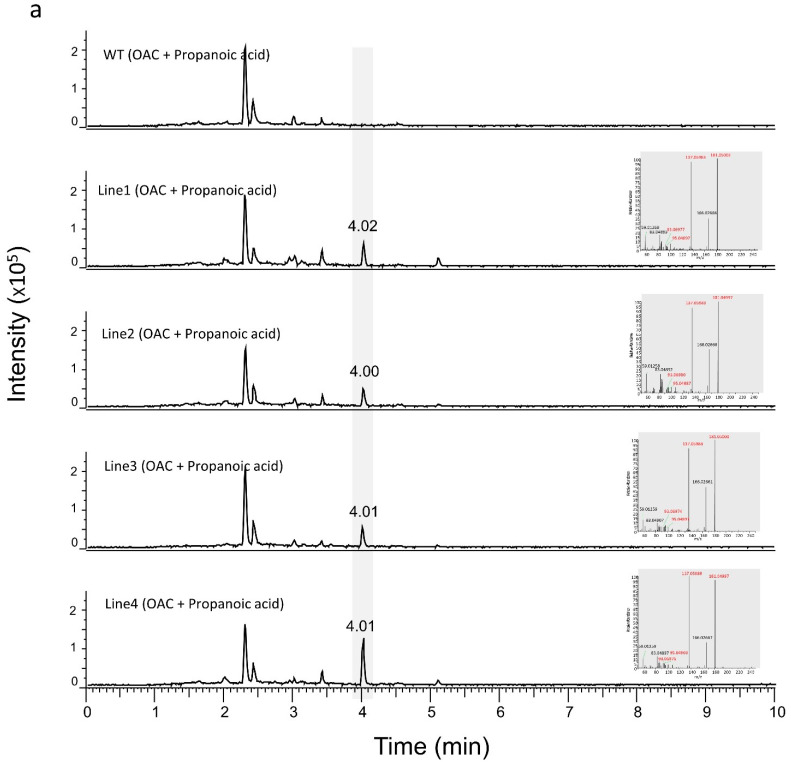

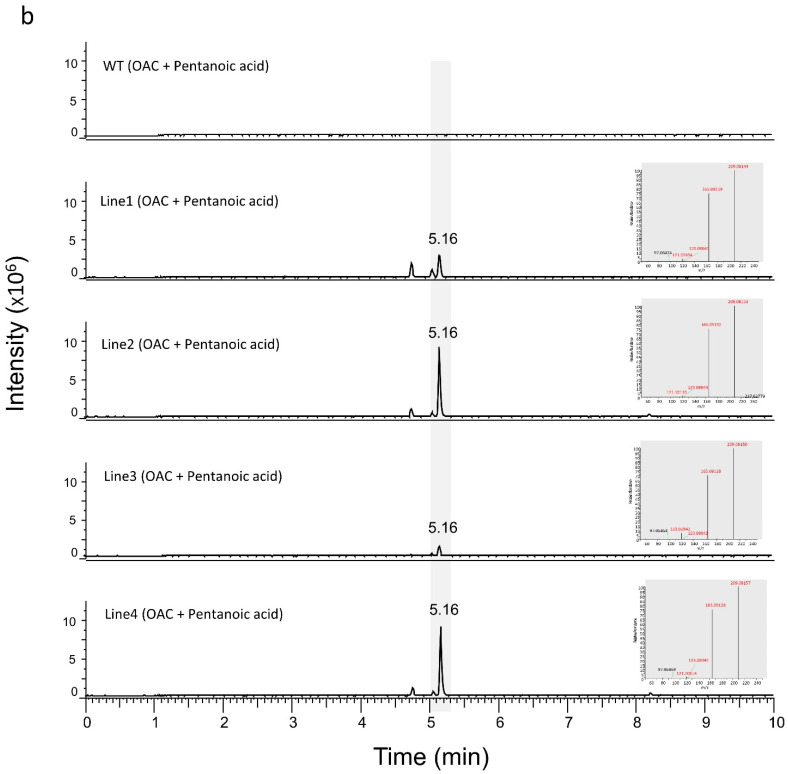

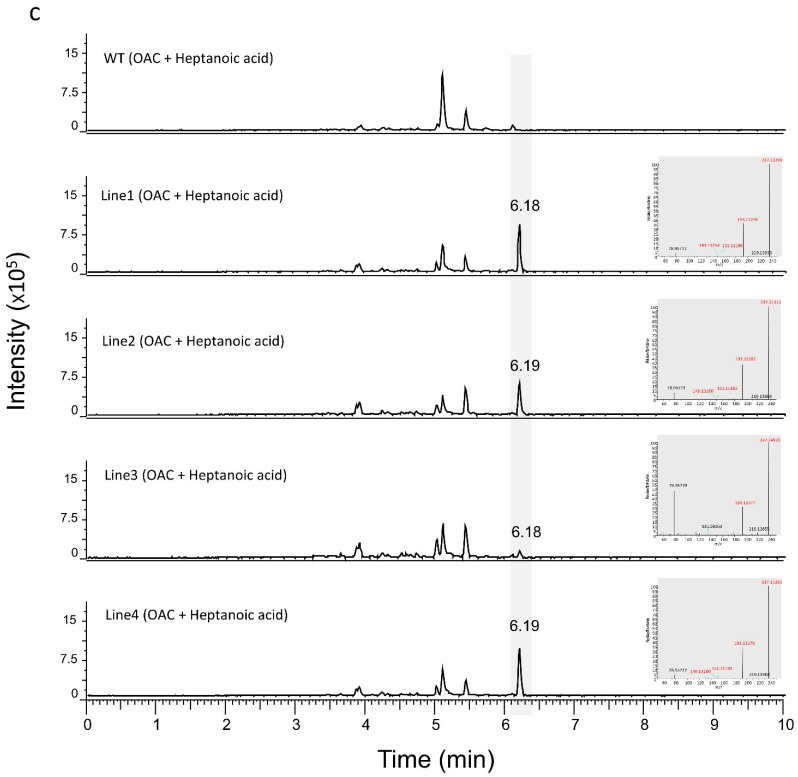
LC-MS results showing the production of products from the transgenic *N. benthamiana* lines infiltrated with *Agrobacterium* cultures of *OAC* and various fatty acids. (**a**) Transgenic *N. benthamiana* lines infiltrated with propanoic acid produced homoorsellinic acid. (**b**) Transgenic *N. benthamiana* lines infiltrated with pentanoic acid produced 2,4-Dihydroxy-6-butylbenzoic acid. (**c**) Transgenic *N. benthamiana* lines infiltrated with heptanoic acid produced 2,4-Dihydroxy-6-hexylbenzoic acid. The signature ions of the representative peaks are shown in red at the right of each chromatogram. *OAC*; *olivetolic acid cyclase*.

Apart from natural cannabinoids, several novel cannabinoid analogues are being investigated for their medicinal properties, as the modifications in analogues can alter the potency and affinity of receptor binding [36]. Specifically, the length of the alkyl side chain, which is an important pharmacophore [37,38], has a direct relation to the receptor binding affinity [39,40]. The length of the cannabinoid alkyl side chain depends on the chain length of the alkylresorcinol fatty acid starter unit [23]. To test whether changing the precursor fatty acids would lead to the production of analogues of cannabinoid precursors, we first infiltrated the leaves of transgenic lines with *Agrobacterium* cultures harbouring *OAC*. After two days, the leaves were infiltrated with a series of fatty acid substrates with varying chain lengths. The list of substrates used, and products formed, can be found in Table S3. As seen in Figure 5a–c, infiltration of propanoic acid, pentanoic acid and heptanoic acid led to the formation of homoorsellinic acid, 2,4-dihydroxy-6-butylbenzoic acid and 2,4-dihydroxy-6-hexylbenzoic acid, respectively, which were identified by their respective precursor and product ions. These products can be used as substrates for the formation of different types of unnatural cannabinoids. 

### 3.3. Production of Cannabigerolic Acid in N. benthamiana Plants

**Figure 6:**
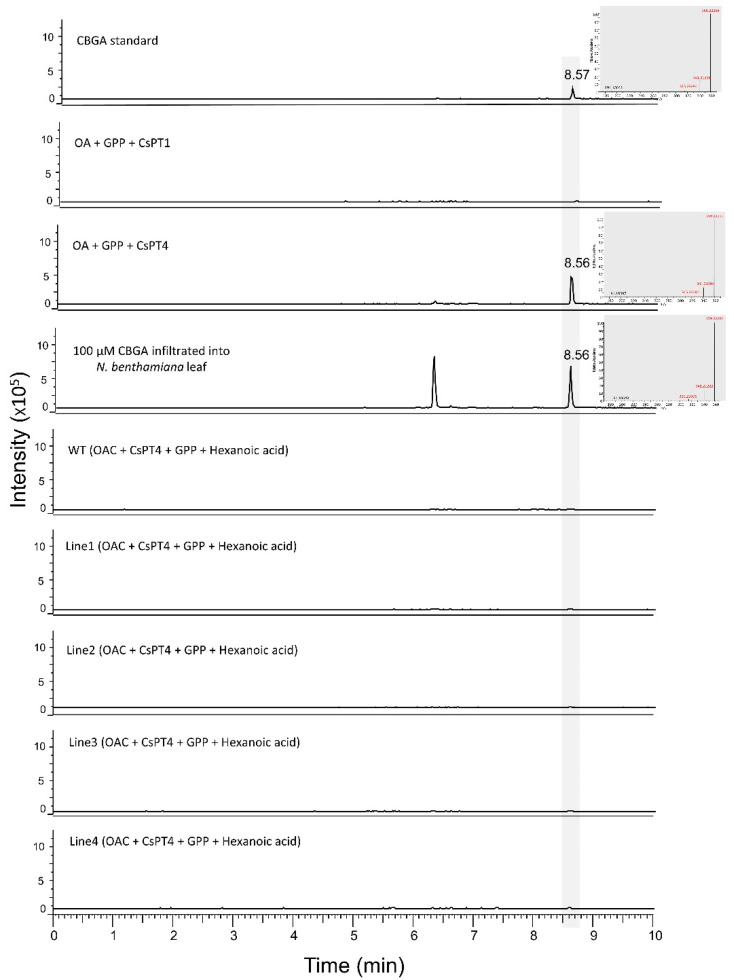
LC-MS results for transgenic and WT *N. benthamiana* leaves transiently expressing *OAC* and *CsPT1*/*CsPT4*. The signature ions of the representative peaks are shown in red at the right of each chromatogram. *OAC*; *olivetolic acid cyclase*, OA; olivetolic acid, GPP; geranyl diphosphate, *CsPT1*; *geranyldiphosphate:olivetolate geranyltransferase1*, *CsPT4*; *geranyldiphosphate:olivetolate geranyltransferase 4*.

CBGA is the acid form of cannabigerol, a non-psychoactive cannabinoid that serves as the precursor for other cannabinoids. Successful production of OA led us to further engineer the cannabinoid pathway to produce CBGA in *N. benthamiana* plants. To test the production of CBGA, two previously identified enzymes, *CsPT1* and *CsPT4* [16], were cloned and expressed in *Agrobacterium*. Initially, to test the enzymatic activities of CsPT1 and CsPT4, *N. benthamiana* leaves were infiltrated with *Agrobacterium* cultures containing *CsPT1* or *CsPT4* and left for two days for transient expression. After two days, the leaves were infiltrated with 100 µM OA + 1 mM GPP and processed for LC-MS analysis after further 24 h incubation. As seen in Figure 6, *N. benthamiana* leaves infiltrated with *CsPT4* were able to produce CBGA, which was absent in leaves expressing *CsPT1*. This confirmed the CBGAS activity of CsPT4, which was then transiently expressed in transgenic *N. benthamiana* plants. After 2 days of infiltration of *CsPT4* and *OAC Agrobacterium* cultures, the leaves were infiltrated with 1 mM GPP + 1 mM hexanoic acid. After 24 h, the leaves were processed for LC-MS analysis. However, no peaks of CBGA were detected in any of the transgenic lines (Figure 6) despite producing OA (Figure S3). The presence of CBGA peaks was analysed by RT of CBGA standard and by the presence of signature ions in the mass spectrum which is specific to CBGA.

### 3.4. Production of Olivetol in N. benthamiana Cell Lines

**Figure 7:**
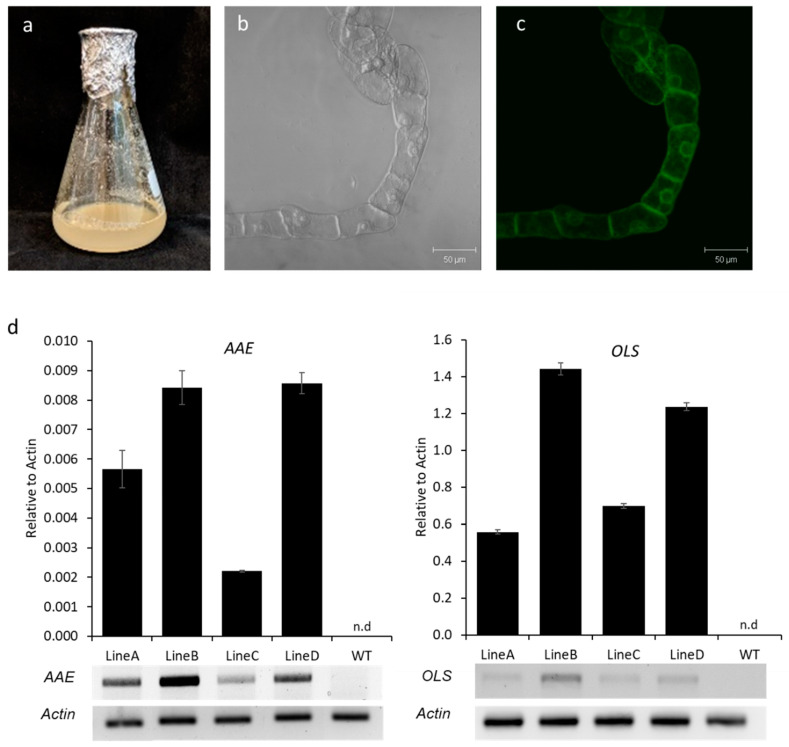
Visual screening and expression analysis of *AAE* and *OLS* in cell lines. (**a**) Representative photo showing suspension culture of *N. benthamiana* cells. Representative confocal image showing transgenic *N. benthamiana*-induced cells under bright field (**b**) and GFP filter (**c**). (**d**) qRT-PCR analysis for the expression of *AAE* and *OLS* in the transgenic (Lines A, B, C, D) and WT *N. benthamiana* cell lines. *Actin* was used as the internal reference. *AAE*; *acyl-activating enzyme*, *OLS*; *olivetol synthase*, n.d; not detected.

**Figure 8:**
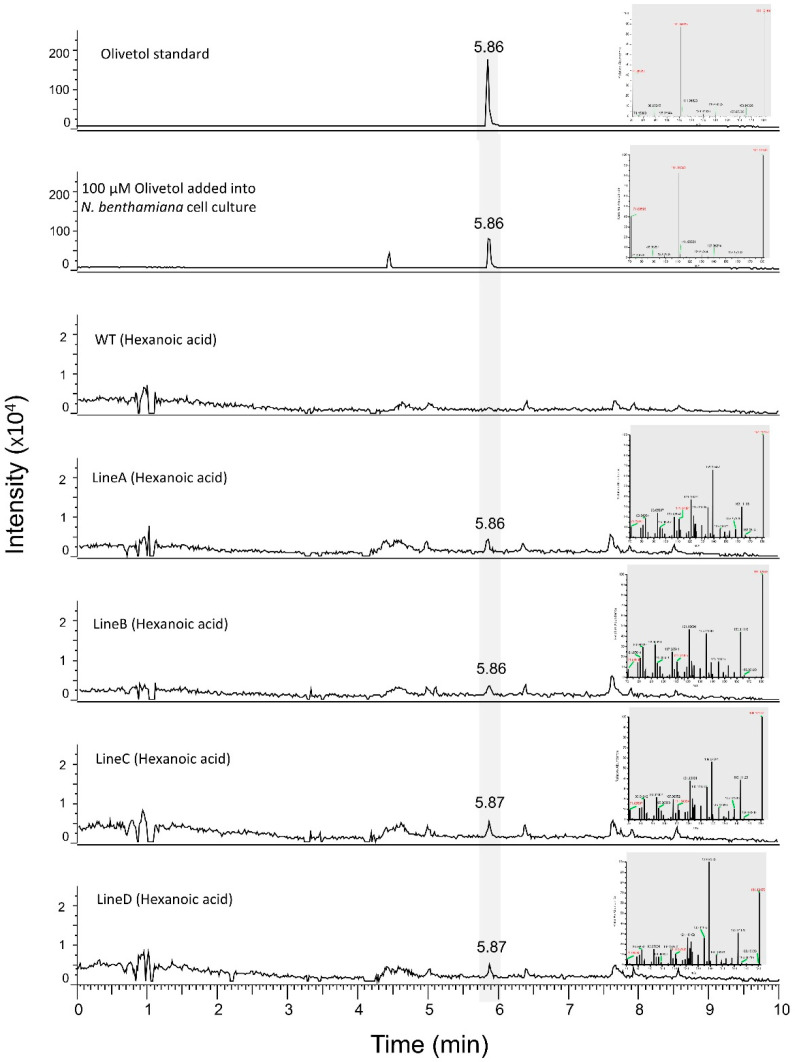
LC-MS results for transgenic and WT *N. benthamiana* cell cultures supplemented with hexanoic acid. A total of 1 mM hexanoic acid substrate was supplemented to the *N. benthamiana* cell cultures for 24 h. Olivetol was produced in the transgenic cell cultures with the same RT as the olivetol standard. The signature ions of the representative peaks are shown in red at the right of each chromatogram.

To test whether cell cultures would be a better platform than transgenics to produce cannabinoids, we developed *N. benthamiana* cell cultures from the transgenic *N. benthamiana* lines. Leaf tissues from transgenic *N. benthamiana* plants were used as explants to initiate callus formation, which was then dispersed into a liquid medium to generate cell suspension. Cells were visually screened for GFP (Figure 7a–c), and expression of *OLS* and *AAE* genes in the cell culture was confirmed by semi-quantitative PCR and qRT-PCR (Figure 7d and Figure S2b). When the cell suspensions were subjected to LC-MS analysis, minute peaks of olivetol were observed (Figure 8). Although a direct comparison to amounts produced in transgenic plants is not possible due to different extraction processes, since very low amounts of olivetol production was observed in cell cultures, we decided to focus on transgenic plants as they appeared to be a better platform to produce cannabinoid precursors when compared to the cell suspension.

## 4. Discussion

Using heterologous systems for producing rare and minor cannabinoids has created new opportunities to explore their therapeutic potential. These heterologous systems will also aid in exploiting the new chemical space to produce novel cannabinoid-like analogues or novel derivatised compounds from existing cannabinoids and investigate them for their bioactivities [16,24,41]. Among the various heterologous systems, *N. benthamiana* is a well-established host system used widely to produce specialised plant metabolites. Specifically, in a recent review on the origins and biosynthesis of phytocannabinoids, *N. benthamiana* was identified as a promising heterologous host to produce cannabinoids in planta due to its high transformation rate, its natural ability to accommodate the supply of precursor GPP, thus aiding in diverting the existing pathway towards the production of cannabinoids, and also because it possesses glandular trichomes that can be utilised to avoid autotoxicity resulting from cannabinoid pathway intermediates [42]. However, attempts to produce cannabinoids in *N. benthamiana* have been limited. Although the use of in vitro plant cell cultures for cannabinoid production has been explored [43], research on the use of *N. benthamiana* cell cultures for cannabinoid production is scarce. Three major upstream genes for building a cannabinoid pathway are *AAE*, *OLS* and *OAC*. Here, we have successfully engineered *N. benthamiana* plants and produced OA and DA, along with different analogues of cannabinoid precursors, by using different fatty acids. This highlights the importance of the use of plant platforms to produce unnatural cannabinoid-like analogues.

As shown in Figure S1a, *AAE* was cloned under the light-inducible promoter AtRBCS2B to avoid over-accumulation of fatty acyl-CoA. *OLS* was cloned under CaMV 35S promoter, which is a constitutive promoter, to ensure continuous expression of the gene to obtain maximum production of cannabinoid precursors. 

When olivetol and divarinol were infiltrated into wild-type *N. benthamiana* plants, we saw a split in the peak and a shift in RT. This can be due to some matrix components loosely binding to the analyte, thus affecting the LC behaviour of the analyte on the column [35]. In case of transgenic plants producing divarinol/DA, additional peaks observed can be due to the expression of enzymes, leading to the formation of other compounds with the same fragmentation properties. Previously, when the cannabinoid biosynthetic enzymes were transiently expressed in *N. benthamiana*, it predominantly led to the formation of OA-glucosides [17]. The authors postulated that uridine diphosphate glucosyltransferases (UGTs) present in *N. benthamiana* can metabolise hydrophobic nonendogenous compounds, thus using OA as a substrate to produce its glucosylated analogue. However, we were able to detect OA in our transgenic tobacco lines and this difference may be due to the lower levels of OA produced due to stable insertion of pathway genes into the genome rather than transient expression [17]. Generally transient expression of recombinant proteins is proposed to be more fast and robust when compared to stable transformation [44]. 

Type III PKS enzymes such as OLS are shown to be highly promiscuous [41]. In our study, as expected, OLS was able to accept n-butyryl-CoA and produce divarinol and DA which has been shown previously [12,16]. Additionally, the infiltration of various fatty acids into the transgenic plants led to the production of analogues of cannabinoid precursors. Previously, yeast has been engineered to produce analogues of cannabinoid precursors [16]. To our knowledge, we are the first to use *N. benthamiana* as a stable heterologous host to produce analogues of cannabinoid precursors. Many cannabinoid analogues are known to act as multi-target drugs for the treatment of several diseases [45]. In this regard, our transgenic plants will be useful for exploring and producing novel cannabinoid analogues.

Though CsPT1 was initially reported to have CBGAS activity, in our experiments it did not show any CBGAS activity. The absence of CBGAS activity by CsPT1 has been previously reported in yeast and *N. benthamiana* as well [16,17]. Moreover, our efforts to produce CBGA in transgenic *N. benthamiana* plants was not successful, despite the successful production of OA and confirming the CBGAS activity of CsPT4. One of the reasons for this might be due to extremely low levels of OA produced in transgenic *N. benthamiana* lines. The highest amount of OA produced in the transgenic *N. benthamiana* plant was ~12 ng/g FW. CBGA formation was observed when 100 µM OA was infiltrated, indicating that if levels of OA production can be elevated by further optimisation, CBGA can be produced in these transgenic plants. Another key challenge for engineering CBGA production in *N. benthamiana* is the toxicity of pathway intermediates. A previous study showed that CBGA can induce cell death via the induction of apoptosis in plant cells [29]. However, in our studies, infiltrating 100 µM of CBGA only caused damage at the site of injection though the solution was spread across the entire leaf (Figure S4). 

Though the *N. benthamiana* plant is known to have a basal capacity for the production of GPP and hexanoic acid [46], in our study we were able to produce cannabinoids only when these precursors were supplied externally. However, these precursor pools may be increased by overexpressing the respective enzymes, which might negate the need to provide precursors externally.

The production of secondary metabolites in cell cultures has a huge benefit because of scaling-up options, leading to mass production and easier extraction when compared to costlier extraction methods from plants. Though a few studies have shown the successful production of cannabinoids in cell suspensions, the yield is very low, and different techniques, such as producing cannabinoids in chloroplasts, or secreting cannabinoids in the culture medium from where they can be easily purified, are still underway [47]. In this study we were also successful in producing olivetol in tobacco cell suspensions. The yields appeared lower when compared to the transgenics, but further comparative analysis needs to be performed. Some reasons for the low production of olivetol might be because of the lesser uptake of hexanoic acid or toxicity arising from the production of olivetol itself. It is shown that CBGA, CBCA and THCA are toxic to cannabis cell suspension cultures [48]. Co-cultivation of CBGA and THCA with *N. benthamiana* cells also caused cell death at 24 h [29]. Hence, cytotoxicity levels of different cannabinoids will be a critical factor that needs to be addressed for further standardization of cannabinoid biosynthesis in cell suspension culture. 

## 5. Conclusions

The production of cannabinoids and their precursors has been achieved in various heterologous hosts [49]. Complete biosynthesis of cannabinoids and their analogues was performed in *Saccharomyces cerevisiae* [16]. Amoeba *Dictyostelium discoideum* was engineered to produce olivetolic acid by expressing the upstream cannabinoid pathway genes [50]. Olivetolic acid has been produced in *Escherichia coli* when supplemented with hexanoic acid [13]. THCA has been produced in *N. benthamiana* root cultures supplemented with CBGA and engineered to express THCAS [28]. A major obstacle in increasing cannabinoid production in a heterologous host is its toxicity. The cannabis plant can avoid the cytotoxicity of cannabinoids by producing them in specialised organs called glandular trichomes. *N. benthamiana* plants have glandular trichomes that can store the toxic cannabinoids which might otherwise damage the cells when produced in yeast or other cell suspensions [43,51,52]. The use of glandular-specific promoters to drive the production in glandular trichomes will help towards increasing yields. However, to date, only transient production of cannabinoids has been carried out in *N. benthamiana* plants. Here, we have successfully developed stable transgenics to produce cannabinoid precursors and established a system that takes advantage of the promiscuity of cannabinoid pathway enzymes to produce different cannabinoid analogues by using different fatty acid precursors. Hence, with further pathway optimisation, the *N. benthamiana* plant is a promising chassis organism to produce existing and novel cannabinoids.

## Data Availability

Sequences of *OLS*, *AAE*, *OAC, CsPT1, CsPT4* and *Actin* are available in the NCBI database under the sequence IDs AB164375.1, JN717233.1, JN679224.1, BK010678.1, BK010648.1, and XM_030632129.1, respectively. Sequences of *TNos* terminator and *AtRBCS2B* promoter are available in File S1.

## Supplementary Materials

The following supporting information can be downloaded at: https://www.mdpi.com/article/10.3390/metabo12121181/s1. File S1: Vector map and sequence details of PK7VRA. Figure S1: Map of PK7VRA vector construction for the synthesis of cannabinoids and Southern blot image showing the number of transgene copies. Figure S2: Dissociation curve analysis. Figure S3: LC-MS results of transgenic *N. benthamiana* lines transiently expressing *OAC* and *CsPT4*. Figure S4: *N. benthamiana* leaf showing damage only at the site of injection of 100 µM of CBGA. Table S1: List of primers used in this study. Table S2: Details of product ions and MS parameters. Table S3: List of substrates used for infiltration and their respective products formed.
