# Supplementary material for: Metabolic Engineering of Nicotiana benthamiana to Produce Cannabinoid Precursors and Their Analogues
Source: Metabolites. 2022 Nov 25;12(12):1181. doi: 10.3390/metabo12121181 (PMC9786632; doi:10.3390/metabo12121181)
Supplement: Supplementary file 1 [file metabolites-12-01181-s001.zip › metabolites-2027773-supplementary.pdf]

Created by GenSmart Design, GenScript

Created time: 20:06:21, 02/18/2021

1. Map

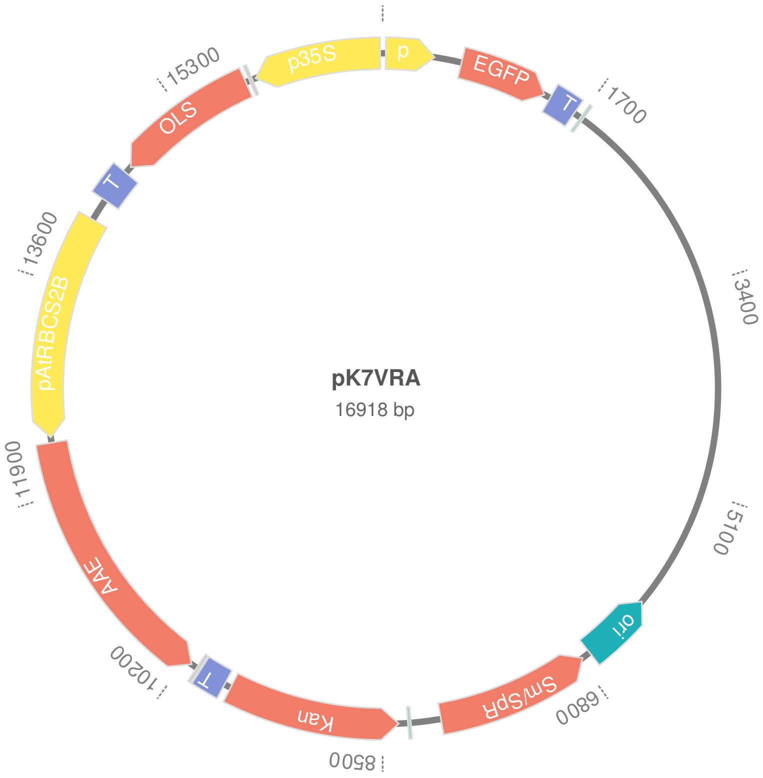

2. Construct Information

Construct name	pK7VRA
Construct size (bp)	16918 bp

3. Sequence

```
AGCTTGCATGCCTGCAGGTTAGCTTGCATGCTGCAGGTCGACTCTCTAGGAATTTGTTCGTGAACTATTAGTTGCGGGCCTTGGCATCCGACTACCTCTGCGGCAATATTATATTCCCTG
TCGAACGTACGGACGTCCAATCGAACGTACGACGTCCAGCTGAGAGATCCTTAAACAAGCACTTGATAATCAACGCCCGGAACCGTAGGCTGATGGAGACGCCGTTATAATATAAGGGAC
p
10 20 30 40 50 60 70 80 90 100 110 120

GGCCCACCGTGAACCCAATTTCGCCTATTTATTCATTACCCCCATTAACATTGAAGTAGTCATGATGGGCCTGCAGCACGTTGGTGAGGCTGGCACAACTCATCCATATACTTTCTGACC
CCGGGTGGCACTTGGGTTAAAGCGGATAAATAAGTAATGGGGGTAATTGTAACTTCATCAGTACTACCCGGACGTCGTGCAACCACTCCGACCGTGTTGAGTAGGTATATGAAAGACTGG
130 140 150 160 170 180 190 200 210 220 230 240

GGATCGGCACATTATTGTAGAAAACGCGGACCCACAGCGCACTTTCCAAAGCGGTGCCGCGTCAGAATGCGCTGGCAGAAAAAAATTAATCCAAAAGTACCCTCCAAGCAGCCCATATAA
CCTAGCCGTGTAATAACATCTTTTGCGCCTGGGTGTCGCGTGAAAGGTTTCGCCACGGCGCAGTCTTACGCGACCGTCTTTTTTTAATTAGGTTTTCATGGGAGGTTCGTCGGGTATATT
250 260 270 280 290 300 310 320 330 340 350 360

ACGCGTTTACAAATCCGCTAACCTCAACAATTTGAGCAGAGAAAATTCGCTAGAGGATCCCCGGGTACCGAGCTCGAATTCTCAACACAACATATACAAAACAAACGAATCTCAAGCAAT
TGCGCAAATGTTTAGGCGATTGGAGTTGTTAAACTCGTCTCTTTTAAGCGATCTCCTAGGGGCCCATGGCTCGAGCTTAAGAGTTGTGTTGTATATGTTTTGTTTGCTTAGAGTTCGTTA
370 380 390 400 410 420 430 440 450 460 470 480

CAAGCATTCTACTTCTATTGCAGCAATTTAAATCATTTCTTTTAAAGCAAAAGCTATTTTCTGAAAATTTTCACCATTTACGAACGATAGCCATGGTGAAGACTAATCTTTTTCTCTTTT
GTTCGTAAGATGAAGATAACGTCGTTAAATTTAGTAAAGAAAATTTCGTTTTCGATAAAAGACTTTTAAAAGTGGTAAATGCTTGCTATCGGTACCACTTCTGATTAGAAAAAGAGAAAA
490 500 510 520 530 540 550 560 570 580 590 600

TCATCTTTTCACTTCTCCTATCATTATCCTCGGCCGACATGGTGAGCAAGGGCGAGGAGCTGTTCACCGGGGTGGTGCCCATCCTGGTCGAGCTGGACGGCGACGTAAACGGCCACAAGT
AGTAGAAAAGTGAAGAGGATAGTAATAGGAGCCGGCTGTACCACTCGTTCCCGCTCCTCGACAAGTGGCCCCACCACGGGTAGGACCAGCTCGACCTGCCGCTGCATTTGCCGGTGTTCA
M V S K G E E L F T G V V P I L V E L D G D V N G H K
EGFP
610 620 630 640 650 660 670 680 690 700 710 720

TCAGCGTGTCCGGCGAGGGCGAGGGCGATGCCACCTACGGCAAGCTGACCCTGAAGTTCATCTGCACCACCGGCAAGCTGCCCGTGCCCTGGCCCACCCTCGTGACCACCCTGACCTACG
AGTCGCACAGGCCGCTCCCGCTCCCGCTACGGTGGATGCCGTTCGACTGGGACTTCAAGTAGACGTGGTGGCCGTTCGACGGGCACGGGACCGGGTGGGAGCACTGGTGGGACTGGATGC
F S V S G E G E G D A T Y G K L T L K F I C T T G K L P V P W P T L V T T L T Y
730 740 750 760 770 780 790 800 810 820 830 840

GCGTGCAGTGCTTCAGCCGCTACCCCGACCACATGAAGCAGCACGACTTCTTCAAGTCCGCCATGCCCGAAGGCTACGTCCAGGAGCGCACCATCTTCTTCAAGGACGACGGCAACTACA
CGCACGTCACGAAGTCGGCGATGGGGCTGGTGTACTTCGTCGTGCTGAAGAAGTTCAGGCGGTACGGGCTTCCGATGCAGGTCCTCGCGTGGTAGAAGAAGTTCCTGCTGCCGTTGATGT
G V Q C F S R Y P D H M K Q H D F F K S A M P E G Y V Q E R T I F F K D D G N Y
850 860 870 880 890 900 910 920 930 940 950 960

AGACCCGCGCCGAGGTGAAGTTCGAGGGCGACACCCTGGTGAACCGCATCGAGCTGAAGGGCATCGACTTCAAGGAGGACGGCAACATCCTGGGGCACAAGCTGGAGTACAACTACAACA
TCTGGGCGCGGCTCCACTTCAAGCTCCCGCTGTGGGACCACTTGGCGTAGCTCGACTTCCCGTAGCTGAAGTTCCTCCTGCCGTTGTAGGACCCCGTGTTCGACCTCATGTTGATGTTGT
K T R A E V K F E G D T L V N R I E L K G I D F K E D G N I L G H K L E Y N Y N
970 980 990 1000 1010 1020 1030 1040 1050 1060 1070 1080

GCCACAACGTCTATATCATGGCCGACAAGCAGAAGAACGGCATCAAGGTGAACTTCAAGATCCGCCACAACATCGAGGACGGCAGCGTGCAGCTCGCCGACCACTACCAGCAGAACACCC
CGGTGTTGCAGATATAGTACCGGCTGTTCGTCTTCTTGCCGTAGTTCCACTTGAAGTTCTAGGCGGTGTTGTAGCTCCTGCCGTCGCACGTCGAGCGGCTGGTGATGGTCGTCTTGTGGG
S H N V Y I M A D K Q K N G I K V N F K I R H N I E D G S V Q L A D H Y Q Q N T
1090 1100 1110 1120 1130 1140 1150 1160 1170 1180 1190 1200

CCATCGGCGACGGCCCCGTGCTGCTGCCCGACAACCACTACCTGAGCACCCAGTCCGCCCTGAGCAAAGACCCCAACGAGAAGCGCGATCACATGGTCCTGCTGGAGTTCGTGACCGCCG
GGTAGCCGCTGCCGGGGCACGACGACGGGCTGTTGGTGATGGACTCGTGGGTCAGGCGGGACTCGTTTCTGGGGTTGCTCTTCGCGCTAGTGTACCAGGACGACCTCAAGCACTGGCGGC
P I G D G P V L L P D N H Y L S T Q S A L S K D P N E K R D H M V L L E F V T A
1210 1220 1230 1240 1250 1260 1270 1280 1290 1300 1310 1320
```

CCGGGATCACTCTCGGCATGGACGAGCTGTACAAGAAGGACGAGCTGTAAGCGCCGCCCGGCTGCAGCCCGGGGGATCCACTAGTTCTAGAGTCCGCAAAAATCACCAGTCTCTCTCTAC
GGCCCTAGTGAGAGCCGTACCTGCTCGACATGTTCTTCCTGCTCGACATTCGCGGCGGGCCGACGTCGGGCCCCCTAGGTGATCAAGATCTCAGGCGTTTTTAGTGGTCAGAGAGAGATG
A G I T L G M D E L Y K
T
10 20 30 40 50 60 70 80 90 100 110 120

AAATCTATCTCTCTCTATTTTTCTCCAGAATAATGTGTGAGTAGTTCCCACATAAGGGAATTAGGGTTCTTATAGGGTTTCGCTCATGTGTTGAGCATATAAGAAACCCTTAGTATGTAT
TTTAGATAGAGAGAGATAAAAAGAGGTCTTATTACACACTCATCAAGGGTGTATTCCCTTAATCCCAAGAATATCCCAAAGCGAGTACACAACTCGTATATTCTTTGGGAATCATACATA
130 140 150 160 170 180 190 200 210 220 230 240

TTGTATTTGTAAAATACTTCTATCAATAAAATTTCTAATTCCTAAAACCAAAATCCAGTGACCTGCAGGCATGCAAGCTTAGCTTGAGCTTGGATCAGATTGTCGTTTCCCGCCTTCAGT
AACATAAACATTTTATGAAGATAGTTATTTTAAAGATTAAGGATTTTGGTTTTAGGTCACTGGACGTCCGTACGTTCGAATCGAACTCGAACCTAGTCTAACAGCAAAGGGCGGAAGTCA
250 260 270 280 290 300 310 320 330 340 350 360

TTAAACTATCAGTGTTTGACAGGATATATTGGCGGGTAAACCTAAGAGAAAAGAGCGTTTATTAGAATAACGGATATTTAAAAGGGCGTGAAAAGGTTTATCCGTTCGTCCATTTGTATG
AATTTGATAGTCACAAACTGTCCTATATAACCGCCCATTTGGATTCTCTTTTCTCGCAAATAATCTTATTGCCTATAAATTTTCCCGCACTTTTCCAAATAGGCAAGCAGGTAAACATAC
RB T-DNA repeat
370 380 390 400 410 420 430 440 450 460 470 480

TGCATGCCAACCACAGGGTTCCCCTCGGGATCAAAGTACTTTGATCCAACCCCTCCGCTGCTATAGTGCAGTCGGCTTCTGACGTTCAGTGCAGCCGTCTTCTGAAAACGACATGTCGCA
ACGTACGGTTGGTGTCCCAAGGGGAGCCCTAGTTTCATGAAACTAGGTTGGGGAGGCGACGATATCACGTCAGCCGAAGACTGCAAGTCACGTCGGCAGAAGACTTTTGCTGTACAGCGT
490 500 510 520 530 540 550 560 570 580 590 600

CAAGTCCTAAGTTACGCGACAGGCTGCCGCCCTGCCCTTTTCCTGGCGTTTTCTTGTCGCGTGTTTTAGTCGCATAAAGTAGAATACTTGCGACTAGAACCGGAGACATTACGCCATGAA
GTTCAGGATTCAATGCGCTGTCCGACGGCGGGACGGGAAAAGGACCGCAAAAGAACAGCGCACAAAATCAGCGTATTTCATCTTATGAACGCTGATCTTGGCCTCTGTAATGCGGTACTT
610 620 630 640 650 660 670 680 690 700 710 720

CAAGAGCGCCGCCGCTGGCCTGCTGGGCTATGCCCGCGTCAGCACCGACGACCAGGACTTGACCAACCAACGGGCCGAACTGCACGCGGCCGGCTGCACCAAGCTGTTTTCCGAGAAGAT
GTTCTCGCGGCGGCGACCGGACGACCCGATACGGGCGCAGTCGTGGCTGCTGGTCCTGAACTGGTTGGTTGCCCGGCTTGACGTGCGCCGGCCGACGTGGTTCGACAAAAGGCTCTTCTA
730 740 750 760 770 780 790 800 810 820 830 840

CACCGGCACCAGGCGCGACCGCCCGGAGCTGGCCAGGATGCTTGACCACCTACGCCCTGGCGACGTTGTGACAGTGACCAGGCTAGACCGCCTGGCCCGCAGCACCCGCGACCTACTGGA
GTGGCCGTGGTCCGCGCTGGCGGGCCTCGACCGGTCCTACGAACTGGTGGATGCGGGACCGCTGCAACACTGTCACTGGTCCGATCTGGCGGACCGGGCGTCGTGGGCGCTGGATGACCT
850 860 870 880 890 900 910 920 930 940 950 960

CATTGCCGAGCGCATCCAGGAGGCCGGCGCGGGCCTGCGTAGCCTGGCAGAGCCGTGGGCCGACACCACCACGCCGGCCGGCCGCATGGTGTTGACCGTGTTCGCCGGCATTGCCGAGTT
GTAACGGCTCGCGTAGGTCCTCCGGCCGCGCCCGGACGCATCGGACCGTCTCGGCACCCGGCTGTGGTGGTGCGGCCGGCCGGCGTACCACAACTGGCACAAGCGGCCGTAACGGCTCAA
970 980 990 1000 1010 1020 1030 1040 1050 1060 1070 1080

CGAGCGTTCCCTAATCATCGACCGCACCCGGAGCGGGCGCGAGGCCGCCAAGGCCCGAGGCGTGAAGTTTGGCCCCCGCCCTACCCTCACCCCGGCACAGATCGCGCACGCCCGCGAGCT
GCTCGCAAGGGATTAGTAGCTGGCGTGGGCCTCGCCCGCGCTCCGGCGGTTCCGGGCTCCGCACTTCAAACCGGGGGCGGGATGGGAGTGGGGCCGTGTCTAGCGCGTGCGGGCGCTCGA
1090 1100 1110 1120 1130 1140 1150 1160 1170 1180 1190 1200

GATCGACCAGGAAGGCCGCACCGTGAAAGAGGCGGCTGCACTGCTTGGCGTGCATCGCTCGACCCTGTACCGCGCACTTGAGCGCAGCGAGGAAGTGACGCCCACCGAGGCCAGGCGGCG
CTAGCTGGTCCTTCCGGCGTGGCACTTTCTCCGCCGACGTGACGAACCGCACGTAGCGAGCTGGGACATGGCGCGTGAACTCGCGTCGCTCCTTCACTGCGGGTGGCTCCGGTCCGCCGC
1210 1220 1230 1240 1250 1260 1270 1280 1290 1300 1310 1320

CGGTGCCTTCCGTGAGGACGCATTGACCGAGGCCGACGCCCTGGCGGCCGCCGAGAATGAACGCCAAGAGGAACAAGCATGAAACCGCACCAGGACGGCCAGGACGAACCGTTTTTCATT
GCCACGGAAGGCACTCCTGCGTAACTGGCTCCGGCTGCGGGACCGCCGGCGGCTCTTACTTGCGGTTCTCCTTGTTCGTACTTTGGCGTGGTCCTGCCGGTCCTGCTTGGCAAAAAGTAA
1330 1340 1350 1360 1370 1380 1390 1400 1410 1420 1430 1440

ACCGAAGAGATCGAGGCGGAGATGATCGCGGCCGGGTACGTGTTCGAGCCGCCCGCGCACGTCTCAACCGTGCGGCTGCATGAAATCCTGGCCGGTTTGTCTGATGCCAAGCTGGCGGCC
TGGCTTCTCTAGCTCCGCCTCTACTAGCGCCGGCCCATGCACAAGCTCGGCGGGCGCGTGCAGAGTTGGCACGCCGACGTACTTTAGGACCGGCCAAACAGACTACGGTTCGACCGCCGG
1450 1460 1470 1480 1490 1500 1510 1520 1530 1540 1550 1560

TGGCCGGCCAGCTTGGCCGCTGAAGAAACCGAGCGCCGCCGTCTAAAAAGGTGATGTGTATTTGAGTAAAACAGCTTGCGTCATGCGGTCGCTGCGTATATGATGCGATGAGTAAATAAA
ACCGGCCGGTCGAACCGGCGACTTCTTTGGCTCGCGGCGGCAGATTTTTCCACTACACATAAACTCATTTTGTCGAACGCAGTACGCCAGCGACGCATATACTACGCTACTCATTTATTT
10 20 30 40 50 60 70 80 90 100 110 120

CAAATACGCAAGGGGAACGCATGAAGGTTATCGCTGTACTTAACCAGAAAGGCGGGTCAGGCAAGACGACCATCGCAACCCATCTAGCCCGCGCCCTGCAACTCGCCGGGGCCGATGTTC
GTTTATGCGTTCCCCTTGCGTACTTCCAATAGCGACATGAATTGGTCTTTCCGCCCAGTCCGTTCTGCTGGTAGCGTTGGGTAGATCGGGCGCGGGACGTTGAGCGGCCCCGGCTACAAG
130 140 150 160 170 180 190 200 210 220 230 240

TGTTAGTCGATTCCGATCCCCAGGGCAGTGCCCGCGATTGGGCGGCCGTGCGGGAAGATCAACCGCTAACCGTTGTCGGCATCGACCGCCCGACGATTGACCGCGACGTGAAGGCCATCG
ACAATCAGCTAAGGCTAGGGGTCCCGTCACGGGCGCTAACCCGCCGGCACGCCCTTCTAGTTGGCGATTGGCAACAGCCGTAGCTGGCGGGCTGCTAACTGGCGCTGCACTTCCGGTAGC
250 260 270 280 290 300 310 320 330 340 350 360

GCCGGCGCGACTTCGTAGTGATCGACGGAGCGCCCCAGGCGGCGGACTTGGCTGTGTCCGCGATCAAGGCAGCCGACTTCGTGCTGATTCCGGTGCAGCCAAGCCCTTACGACATATGGG
CGGCCGCGCTGAAGCATCACTAGCTGCCTCGCGGGGTCCGCCGCCTGAACCGACACAGGCGCTAGTTCCGTCGGCTGAAGCACGACTAAGGCCACGTCGGTTCGGGAATGCTGTATACCC
370 380 390 400 410 420 430 440 450 460 470 480

CCACCGCCGACCTGGTGGAGCTGGTTAAGCAGCGCATTGAGGTCACGGATGGAAGGCTACAAGCGGCCTTTGTCGTGTCGCGGGCGATCAAAGGCACGCGCATCGGCGGTGAGGTTGCCG
GGTGGCGGCTGGACCACCTCGACCAATTCGTCGCGTAACTCCAGTGCCTACCTTCCGATGTTCGCCGGAAACAGCACAGCGCCCGCTAGTTTCCGTGCGCGTAGCCGCCACTCCAACGGC
490 500 510 520 530 540 550 560 570 580 590 600

AGGCGCTGGCCGGGTACGAGCTGCCCATTCTTGAGTCCCGTATCACGCAGCGCGTGAGCTACCCAGGCACTGCCGCCGCCGGCACAACCGTTCTTGAATCAGAACCCGAGGGCGACGCTG
TCCGCGACCGGCCCATGCTCGACGGGTAAGAACTCAGGGCATAGTGCGTCGCGCACTCGATGGGTCCGTGACGGCGGCGGCCGTGTTGGCAAGAACTTAGTCTTGGGCTCCCGCTGCGAC
610 620 630 640 650 660 670 680 690 700 710 720

CCCGCGAGGTCCAGGCGCTGGCCGCTGAAATTAAATCAAAACTCATTTGAGTTAATGAGGTAAAGAGAAAATGAGCAAAAGCACAAACACGCTAAGTGCCGGCCGTCCGAGCGCACGCAG
GGGCGCTCCAGGTCCGCGACCGGCGACTTTAATTTAGTTTTGAGTAAACTCAATTACTCCATTTCTCTTTTACTCGTTTTCGTGTTTGTGCGATTCACGGCCGGCAGGCTCGCGTGCGTC
730 740 750 760 770 780 790 800 810 820 830 840

CAGCAAGGCTGCAACGTTGGCCAGCCTGGCAGACACGCCAGCCATGAAGCGGGTCAACTTTCAGTTGCCGGCGGAGGATCACACCAAGCTGAAGATGTACGCGGTACGCCAAGGCAAGAC
GTCGTTCCGACGTTGCAACCGGTCGGACCGTCTGTGCGGTCGGTACTTCGCCCAGTTGAAAGTCAACGGCCGCCTCCTAGTGTGGTTCGACTTCTACATGCGCCATGCGGTTCCGTTCTG
850 860 870 880 890 900 910 920 930 940 950 960

CATTACCGAGCTGCTATCTGAATACATCGCGCAGCTACCAGAGTAAATGAGCAAATGAATAAATGAGTAGATGAATTTTAGCGGCTAAAGGAGGCGGCATGGAAAATCAAGAACAACCAG
GTAATGGCTCGACGATAGACTTATGTAGCGCGTCGATGGTCTCATTTACTCGTTTACTTATTTACTCATCTACTTAAAATCGCCGATTTCCTCCGCCGTACCTTTTAGTTCTTGTTGGTC
970 980 990 1000 1010 1020 1030 1040 1050 1060 1070 1080

GCACCGACGCCGTGGAATGCCCCATGTGTGGAGGAACGGGCGGTTGGCCAGGCGTAAGCGGCTGGGTTGTCTGCCGGCCCTGCAATGGCACTGGAACCCCCAAGCCCGAGGAATCGGCGT
CGTGGCTGCGGCACCTTACGGGGTACACACCTCCTTGCCCGCCAACCGGTCCGCATTCGCCGACCCAACAGACGGCCGGGACGTTACCGTGACCTTGGGGGTTCGGGCTCCTTAGCCGCA
1090 1100 1110 1120 1130 1140 1150 1160 1170 1180 1190 1200

GACGGTCGCAAACCATCCGGCCCGGTACAAATCGGCGCGGCGCTGGGTGATGACCTGGTGGAGAAGTTGAAGGCCGCGCAGGCCGCCCAGCGGCAACGCATCGAGGCAGAAGCACGCCCC
CTGCCAGCGTTTGGTAGGCCGGGCCATGTTTAGCCGCGCCGCGACCCACTACTGGACCACCTCTTCAACTTCCGGCGCGTCCGGCGGGTCGCCGTTGCGTAGCTCCGTCTTCGTGCGGGG
1210 1220 1230 1240 1250 1260 1270 1280 1290 1300 1310 1320

GGTGAATCGTGGCAAGCGGCCGCTGATCGAATCCGCAAAGAATCCCGGCAACCGCCGGCAGCCGGTGCGCCGTCGATTAGGAAGCCGCCCAAGGGCGACGAGCAACCAGATTTTTTCGTT
CCACTTAGCACCGTTCGCCGGCGACTAGCTTAGGCGTTTCTTAGGGCCGTTGGCGGCCGTCGGCCACGCGGCAGCTAATCCTTCGGCGGGTTCCCGCTGCTCGTTGGTCTAAAAAAGCAA
1330 1340 1350 1360 1370 1380 1390 1400 1410 1420 1430 1440

CCGATGCTCTATGACGTGGGCACCCGCGATAGTCGCAGCATCATGGACGTGGCCGTTTTCCGTCTGTCGAAGCGTGACCGACGAGCTGGCGAGGTGATCCGCTACGAGCTTCCAGACGGG
GGCTACGAGATACTGCACCCGTGGGCGCTATCAGCGTCGTAGTACCTGCACCGGCAAAAGGCAGACAGCTTCGCACTGGCTGCTCGACCGCTCCACTAGGCGATGCTCGAAGGTCTGCCC
1450 1460 1470 1480 1490 1500 1510 1520 1530 1540 1550 1560

CACGTAGAGGTTTCCGCAGGGCCGGCCGGCATGGCCAGTGTGTGGGATTACGACCTGGTACTGATGGCGGTTTCCCATCTAACCGAATCCATGAACCGATACCGGGAAGGGAAGGGAGAC
GTGCATCTCCAAAGGCGTCCCGGCCGGCCGTACCGGTCACACACCCTAATGCTGGACCATGACTACCGCCAAAGGGTAGATTGGCTTAGGTACTTGGCTATGGCCCTTCCCTTCCCTCTG
1570 1580 1590 1600 1610 1620 1630 1640 1650 1660 1670 1680

AAGCCCGGCCGCGTGTTCCGTCCACACGTTGCGGACGTACTCAAGTTCTGCCGGCGAGCCGATGGCGGAAAGCAGAAAGACGACCTGGTAGAAACCTGCATTCGGTTAAACACCACGCAC
TTCGGGCCGGCGCACAAGGCAGGTGTGCAACGCCTGCATGAGTTCAAGACGGCCGCTCGGCTACCGCCTTTCGTCTTTCTGCTGGACCATCTTTGGACGTAAGCCAATTTGTGGTGCGTG
10 20 30 40 50 60 70 80 90 100 110 120

GTTGCCATGCAGCGTACGAAGAAGGCCAAGAACGGCCGCCTGGTGACGGTATCCGAGGGTGAAGCCTTGATTAGCCGCTACAAGATCGTAAAGAGCGAAACCGGGCGGCCGGAGTACATC
CAACGGTACGTCGCATGCTTCTTCCGGTTCTTGCCGGCGGACCACTGCCATAGGCTCCCACTTCGGAACTAATCGGCGATGTTCTAGCATTTCTCGCTTTGGCCCGCCGGCCTCATGTAG
130 140 150 160 170 180 190 200 210 220 230 240

GAGATCGAGCTAGCTGATTGGATGTACCGCGAGATCACAGAAGGCAAGAACCCGGACGTGCTGACGGTTCACCCCGATTACTTTTTGATCGATCCCGGCATCGGCCGTTTTCTCTACCGC
CTCTAGCTCGATCGACTAACCTACATGGCGCTCTAGTGTCTTCCGTTCTTGGGCCTGCACGACTGCCAAGTGGGGCTAATGAAAAACTAGCTAGGGCCGTAGCCGGCAAAAGAGATGGCG
250 260 270 280 290 300 310 320 330 340 350 360

CTGGCACGCCGCGCCGCAGGCAAGGCAGAAGCCAGATGGTTGTTCAAGACGATCTACGAACGCAGTGGCAGCGCCGGAGAGTTCAAGAAGTTCTGTTTCACCGTGCGCAAGCTGATCGGG
GACCGTGCGGCGCGGCGTCCGTTCCGTCTTCGGTCTACCAACAAGTTCTGCTAGATGCTTGCGTCACCGTCGCGGCCTCTCAAGTTCTTCAAGACAAAGTGGCACGCGTTCGACTAGCCC
370 380 390 400 410 420 430 440 450 460 470 480

TCAAATGACCTGCCGGAGTACGATTTGAAGGAGGAGGCGGGGCAGGCTGGCCCGATCCTAGTCATGCGCTACCGCAACCTGATCGAGGGCGAAGCATCCGCCGGTTCCTAATGTACGGAG
AGTTTACTGGACGGCCTCATGCTAAACTTCCTCCTCCGCCCCGTCCGACCGGGCTAGGATCAGTACGCGATGGCGTTGGACTAGCTCCCGCTTCGTAGGCGGCCAAGGATTACATGCCTC
490 500 510 520 530 540 550 560 570 580 590 600

CAGATGCTAGGGCAAATTGCCCTAGCAGGGGAAAAAGGTCGAAAAGGTCTCTTTCCTGTGGATAGCACGTACATTGGGAACCCAAAGCCGTACATTGGGAACCGGAACCCGTACATTGGG
GTCTACGATCCCGTTTAACGGGATCGTCCCCTTTTTCCAGCTTTTCCAGAGAAAGGACACCTATCGTGCATGTAACCCTTGGGTTTCGGCATGTAACCCTTGGCCTTGGGCATGTAACCC
610 620 630 640 650 660 670 680 690 700 710 720

AACCCAAAGCCGTACATTGGGAACCGGTCACACATGTAAGTGACTGATATAAAAGAGAAAAAAGGCGATTTTTCCGCCTAAAACTCTTTAAAACTTATTAAAACTCTTAAAACCCGCCTG
TTGGGTTTCGGCATGTAACCCTTGGCCAGTGTGTACATTCACTGACTATATTTTCTCTTTTTTCCGCTAAAAAGGCGGATTTTGAGAAATTTTGAATAATTTTGAGAATTTTGGGCGGAC
730 740 750 760 770 780 790 800 810 820 830 840

GCCTGTGCATAACTGTCTGGCCAGCGCACAGCCGAAGAGCTGCAAAAAGCGCCTACCCTTCGGTCGCTGCGCTCCCTACGCCCCGCCGCTTCGCGTCGGCCTATCGCGGCCGCTGGCCGC
CGGACACGTATTGACAGACCGGTCGCGTGTCGGCTTCTCGACGTTTTTCGCGGATGGGAAGCCAGCGACGCGAGGGATGCGGGGCGGCGAAGCGCAGCCGGATAGCGCCGGCGACCGGCG
850 860 870 880 890 900 910 920 930 940 950 960

TCAAAAATGGCTGGCCTACGGCCAGGCAATCTACCAGGGCGCGGACAAGCCGCGCCGTCGCCACTCGACCGCCGGCGCCCACATCAAGGCACCCTGCCTCGCGCGTTTCGGTGATGACGG
AGTTTTTACCGACCGGATGCCGGTCCGTTAGATGGTCCCGCGCCTGTTCGGCGCGGCAGCGGTGAGCTGGCGGCCGCGGGTGTAGTTCCGTGGGACGGAGCGCGCAAAGCCACTACTGCC
970 980 990 1000 1010 1020 1030 1040 1050 1060 1070 1080

TGAAAACCTCTGACACATGCAGCTCCCGGAGACGGTCACAGCTTGTCTGTAAGCGGATGCCGGGAGCAGACAAGCCCGTCAGGGCGCGTCAGCGGGTGTTGGCGGGTGTCGGGGCGCAGC
ACTTTTGGAGACTGTGTACGTCGAGGGCCTCTGCCAGTGTCGAACAGACATTCGCCTACGGCCCTCGTCTGTTCGGGCAGTCCCGCGCAGTCGCCCACAACCGCCCACAGCCCCGCGTCG
1090 1100 1110 1120 1130 1140 1150 1160 1170 1180 1190 1200

CATGACCCAGTCACGTAGCGATAGCGGAGTGTATACTGGCTTAACTATGCGGCATCAGAGCAGATTGTACTGAGAGTGCACCATATGCGGTGTGAAATACCGCACAGATGCGTAAGGAGA
GTACTGGGTCAGTGCATCGCTATCGCCTCACATATGACCGAATTGATACGCCGTAGTCTCGTCTAACATGACTCTCACGTGGTATACGCCACACTTTATGGCGTGTCTACGCATTCCTCT
1210 1220 1230 1240 1250 1260 1270 1280 1290 1300 1310 1320

AAATACCGCATCAGGCGCTCTTCCGCTTCCTCGCTCACTGACTCGCTGCGCTCGGTCGTTCGGCTGCGGCGAGCGGTATCAGCTCACTCAAAGGCGGTAATACGGTTATCCACAGAATCA
TTTATGGCGTAGTCCGCGAGAAGGCGAAGGAGCGAGTGACTGAGCGACGCGAGCCAGCAAGCCGACGCCGCTCGCCATAGTCGAGTGAGTTTCCGCCATTATGCCAATAGGTGTCTTAGT
1330 1340 1350 1360 1370 1380 1390 1400 1410 1420 1430 1440

GGGGATAACGCAGGAAAGAACATGTGAGCAAAAGGCCAGCAAAAGGCCAGGAACCGTAAAAAGGCCGCGTTGCTGGCGTTTTTCCATAGGCTCCGCCCCCCTGACGAGCATCACAAAAAT
CCCCTATTGCGTCCTTTCTTGTACACTCGTTTTCCGGTCGTTTTCCGGTCCTTGGCATTTTTCCGGCGCAACGACCGCAAAAAGGTATCCGAGGCGGGGGGACTGCTCGTAGTGTTTTTA
ori
1450 1460 1470 1480 1490 1500 1510 1520 1530 1540 1550 1560

CGACGCTCAAGTCAGAGGTGGCGAAACCCGACAGGACTATAAAGATACCAGGCGTTTCCCCCTGGAAGCTCCCTCGTGCGCTCTCCTGTTCCGACCCTGCCGCTTACCGGATACCTGTCC
GCTGCGAGTTCAGTCTCCACCGCTTTGGGCTGTCCTGATATTTCTATGGTCCGCAAAGGGGGACCTTCGAGGGAGCACGCGAGAGGACAAGGCTGGGACGGCGAATGGCCTATGGACAGG
1570 1580 1590 1600 1610 1620 1630 1640 1650 1660 1670 1680

GCCTTTCTCCCTTCGGGAAGCGTGGCGCTTTCTCATAGCTCACGCTGTAGGTATCTCAGTTCGGTGTAGGTCGTTCGCTCCAAGCTGGGCTGTGTGCACGAACCCCCCGTTCAGCCCGAC
CGGAAAGAGGGAAGCCCTTCGCACCGCGAAAGAGTATCGAGTGCGACATCCATAGAGTCAAGCCACATCCAGCAAGCGAGGTTCGACCCGACACACGTGCTTGGGGGGCAAGTCGGGCTG

10 20 30 40 50 60 70 80 90 100 110 120

CGCTGCGCCTTATCCGGTAACTATCGTCTTGAGTCCAACCCGGTAAGACACGACTTATCGCCACTGGCAGCAGCCACTGGTAACAGGATTAGCAGAGCGAGGTATGTAGGCGGTGCTACA
GCGACGCGGAATAGGCCATTGATAGCAGAACTCAGGTTGGGCCATTCTGTGCTGAATAGCGGTGACCGTCGTCGGTGACCATTGTCCTAATCGTCTCGCTCCATACATCCGCCACGATGT

130 140 150 160 170 180 190 200 210 220 230 240

GAGTTCTTGAAGTGGTGGCCTAACTACGGCTACACTAGAAGGACAGTATTTGGTATCTGCGCTCTGCTGAAGCCAGTTACCTTCGGAAAAAGAGTTGGTAGCTCTTGATCCGGCAAACAA
CTCAAGAACTTCACCACCGGATTGATGCCGATGTGATCTTCCTGTCATAAACCATAGACGCGAGACGACTTCGGTCAATGGAAGCCTTTTTCTCAACCATCGAGAACTAGGCCGTTTGTT

250 260 270 280 290 300 310 320 330 340 350 360

ACCACCGCTGGTAGCGGTGGTTTTTTTGTTTGCAAGCAGCAGATTACGCGCAGAAAAAAAGGATCTCAAGAAGATCCTTTGATCTTTTCTACGGGGTCTGACGCTCAGTGGAACGAAAAC
TGGTGGCGACCATCGCCACCAAAAAAACAAACGTTCGTCGTCTAATGCGCGTCTTTTTTTCCTAGAGTTCTTCTAGGAAACTAGAAAAGATGCCCCAGACTGCGAGTCACCTTGCTTTTG

370 380 390 400 410 420 430 440 450 460 470 480

TCACGTTAAGGGATTTTGGTCATGCATGATATATCTCCCAATTTGTGTAGGGCTTATTATGCACGCTTAAAAATAATAAAAGCAGACTTGACCTGATAGTTTGGCTGTGAGCAATTATGT
AGTGCAATTCCCTAAAACCAGTACGTACTATATAGAGGGTTAAACACATCCCGAATAATACGTGCGAATTTTTATTATTTTCGTCTGAACTGGACTATCAAACCGACACTCGTTAATACA

Sm/SpR

490 500 510 520 530 540 550 560 570 580 590 600

GCTTAGTGCATCTAATCGCTTGAGTTAACGCCGGCGAAGCGGCGTCGGCTTGAACGAATTTCTAGCTAGACATTATTTGCCGACTACCTTGGTGATCTCGCCTTTCACGTAGTGGACAAA
CGAATCACGTAGATTAGCGAACTCAATTGCGGCCGCTTCGCCGCAGCCGAACTTGCTTAAAGATCGATCTGTAATAAACGGCTGATGGAACCACTAGAGCGGAAAGTGCATCACCTGTTT

610 620 630 640 650 660 670 680 690 700 710 720

TTCTTCCAACTGATCTGCGCGCGAGGCCAAGCGATCTTCTTCTTGTCCAAGATAAGCCTGTCTAGCTTCAAGTATGACGGGCTGATACTGGGCCGGCAGGCGCTCCATTGCCCAGTCGGC
AAGAAGGTTGACTAGACGCGCGCTCCGGTTCGCTAGAAGAAGAACAGGTTCTATTCGGACAGATCGAAGTTCATACTGCCCGACTATGACCCGGCCGTCCGCGAGGTAACGGGTCAGCCG

730 740 750 760 770 780 790 800 810 820 830 840

AGCGACATCCTTCGGCGCGATTTTGCCGGTTACTGCGCTGTACCAAATGCGGGACAACGTAAGCACTACATTTCGCTCATCGCCAGCCCAGTCGGGCGGCGAGTTCCATAGCGTTAAGGT
TCGCTGTAGGAAGCCGCGCTAAAACGGCCAATGACGCGACATGGTTTACGCCCTGTTGCATTCGTGATGTAAAGCGAGTAGCGGTCGGGTCAGCCCGCCGCTCAAGGTATCGCAATTCCA

850 860 870 880 890 900 910 920 930 940 950 960

TTCATTTAGCGCCTCAAATAGATCCTGTTCAGGAACCGGATCAAAGAGTTCCTCCGCCGCTGGACCTACCAAGGCAACGCTATGTTCTCTTGCTTTTGTCAGCAAGATAGCCAGATCAAT
AAGTAAATCGCGGAGTTTATCTAGGACAAGTCCTTGGCCTAGTTTCTCAAGGAGGCGGCGACCTGGATGGTTCCGTTGCGATACAAGAGAACGAAAACAGTCGTTCTATCGGTCTAGTTA

970 980 990 1000 1010 1020 1030 1040 1050 1060 1070 1080

GTCGATCGTGGCTGGCTCGAAGATACCTGCAAGAATGTCATTGCGCTGCCATTCTCCAAATTGCAGTTCGCGCTTAGCTGGATAACGCCACGGAATGATGTCGTCGTGCACAACAATGGT
CAGCTAGCACCGACCGAGCTTCTATGGACGTTCTTACAGTAACGCGACGGTAAGAGGTTTAACGTCAAGCGCGAATCGACCTATTGCGGTGCCTTACTACAGCAGCACGTGTTGTTACCA

1090 1100 1110 1120 1130 1140 1150 1160 1170 1180 1190 1200

GACTTCTACAGCGCGGAGAATCTCGCTCTCTCCAGGGGAAGCCGAAGTTTCCAAAAGGTCGTTGATCAAAGCTCGCCGCGTTGTTTCATCAAGCCTTACGGTCACCGTAACCAGCAAATC
CTGAAGATGTCGCGCCTCTTAGAGCGAGAGAGGTCCCCTTCGGCTTCAAAGGTTTTCCAGCAACTAGTTTCGAGCGGCGCAACAAAGTAGTTCGGAATGCCAGTGGCATTGGTCGTTTAG

1210 1220 1230 1240 1250 1260 1270 1280 1290 1300 1310 1320

AATATCACTGTGTGGCTTCAGGCCGCCATCCACTGCGGAGCCGTACAAATGTACGGCCAGCAACGTCGGTTCGAGATGGCGCTCGATGACGCCAACTACCTCTGATAGTTGAGTCGATAC
TTATAGTGACACACCGAAGTCCGGCGGTAGGTGACGCCTCGGCATGTTTACATGCCGGTCGTTGCAGCCAAGCTCTACCGCGAGCTACTGCGGTTGATGGAGACTATCAACTCAGCTATG

10 20 30 40 50 60 70 80 90 100 110 120

TTCGGCGATCACCGCTTCCCCCATGATGTTTAACTTTGTTTTAGGGCGACTGCCCTGCTGCGTAACATCGTTGCTGCTCCATAACATCAAACATCGACCCACGGCGTAACGCGCTTGCTG
AAGCCGCTAGTGGCGAAGGGGGTACTACAAATTGAAACAAAATCCCGCTGACGGGACGACGCATTGTAGCAACGACGAGGTATTGTAGTTTGTAGCTGGGTGCCGCATTGCGCGAACGAC

130 140 150 160 170 180 190 200 210 220 230 240

CTTGGATGCCCGAGGCATAGACTGTACCCCAAAAAAACATGTCATAACAAGAAGCCATGAAAACCGCCACTGCGCCGTTACCACCGCTGCGTTCGGTCAAGGTTCTGGACCAGTTGCGTG
GAACCTACGGGCTCCGTATCTGACATGGGGTTTTTTTGTACAGTATTGTTCTTCGGTACTTTTGGCGGTGACGCGGCAATGGTGGCGACGCAAGCCAGTTCCAAGACCTGGTCAACGCAC

250 260 270 280 290 300 310 320 330 340 350 360

ACGGCAGTTACGCTACTTGCATTACAGCTTACGAACCGAACGAGGCTTATGTCCACTGGGTTCGTGCCCGAATTGATCACAGGCAGCAACGCTCTGTCATCGTTACAATCAACATGCTAC
TGCCGTCAATGCGATGAACGTAATGTCGAATGCTTGGCTTGCTCCGAATACAGGTGACCCAAGCACGGGCTTAACTAGTGTCCGTCGTTGCGAGACAGTAGCAATGTTAGTTGTACGATG

370 380 390 400 410 420 430 440 450 460 470 480

CCTCCGCGAGATCATCCGTGTTTCAAACCCGGCAGCTTAGTTGCCGTTCTTCCGAATAGCATCGGTAACATGAGCAAAGTCTGCCGCCTTACAACGGCTCTCCCGCTGACGCCGTCCCGG
GGAGGCGCTCTAGTAGGCACAAAGTTTGGGCCGTCGAATCAACGGCAAGAAGGCTTATCGTAGCCATTGTACTCGTTTCAGACGGCGGAATGTTGCCGAGAGGGCGACTGCGGCAGGGCC

490 500 510 520 530 540 550 560 570 580 590 600

ACTGATGGGCTGCCTGTATCGAGTGGTGATTTTGTGCCGAGCTGCCGGTCGGGGAGCTGTTGGCTGGCTGGTGGCAGGATATATTGTGGTGTAAACAAATTGACGCTTAGACAACTTAAT
TGACTACCCGACGGACATAGCTCACCACTAAAACACGGCTCGACGGCCAGCCCCTCGACAACCGACCGACCACCGTCCTATATAACACCACATTTGTTTAACTGCGAATCTGTTGAATTA

LB T-DNA repeat

610 620 630 640 650 660 670 680 690 700 710 720

AACACATTGCGGACGTTTTTAATGTACTGAATTAACGCCGAATTGAATTATCAGCTTGCATGCCGGTCGATCTAGTAACATAGATGACACCGCGCGCGATAATTTATCCTAGTTTGCGCG
TTGTGTAACGCCTGCAAAAATTACATGACTTAATTGCGGCTTAACTTAATAGTCGAACGTACGGCCAGCTAGATCATTGTATCTACTGTGGCGCGCGCTATTAAATAGGATCAAACGCGC

Kan

730 740 750 760 770 780 790 800 810 820 830 840

CTATATTTTGTTTTCTATCGCGTATTAAATGTATAATTGCGGGACTCTAATCATAAAAACCCATCTCATAAATAACGTCATGCATTACATGTTAATTATTACATGCTTAACGTAATTCAA
GATATAAAACAAAAGATAGCGCATAATTTACATATTAACGCCCTGAGATTAGTATTTTTGGGTAGAGTATTTATTGCAGTACGTAATGTACAATTAATAATGTACGAATTGCATTAAGTT

850 860 870 880 890 900 910 920 930 940 950 960

CAGAAATTATATGATAATCATCGCAAGACCGGCAACAGGATTCAATCTTAAGAAACTTTATTGCCAAATGTTTGAACGATCTGCTTGACTCTAGCTAGAGTCCGAACCCCAGAGTCCCGC
GTCTTTAATATACTATTAGTAGCGTTCTGGCCGTTGTCCTAAGTTAGAATTCTTTGAAATAACGGTTTACAAACTTGCTAGACGAACTGAGATCGATCTCAGGCTTGGGGTCTCAGGGCG

970 980 990 1000 1010 1020 1030 1040 1050 1060 1070 1080

TCAGGAAGAACTCGTCAAGAAGGCGATAGAAGGCGATGCGCTGCGAATCGGGAGCGGCGATACCGTAAAGCACGAGGAAGCGGTCAGCCCATTCGCCGCCAAGCTCTTCAGCAATATCAC
AGTCCTTCTTGAGCAGTTCTTCCGCTATCTTCCGCTACGCGACGCTTAGCCCTCGCCGCTATGGCATTTCGTGCTCCTTCGCCAGTCGGGTAAGCGGCGGTTCGAGAAGTCGTTATAGTG

1090 1100 1110 1120 1130 1140 1150 1160 1170 1180 1190 1200

GGGTAGCCAACGCTATGTCCTGATAGCGGTCCGCCACACCCAGCCGGCCACAGTCGATGAATCCAGAAAAGCGGCCATTTTCCACCATGATATTCGGCAAGCAGGCATCGCCGTGGGTCA
CCCATCGGTTGCGATACAGGACTATCGCCAGGCGGTGTGGGTCGGCCGGTGTCAGCTACTTAGGTCTTTTCGCCGGTAAAAGGTGGTACTATAAGCCGTTCGTCCGTAGCGGCACCCAGT

1210 1220 1230 1240 1250 1260 1270 1280 1290 1300 1310 1320

CGACGAGATCCTCGCCGTCGGGCATCCGCGCCTTGAGCCTGGCGAACAGTTCGGCTGGCGCGAGCCCCTGATGCTCTTCGTCCAGATCATCCTGATCGACAAGACCGGCTTCCATCCGAG
GCTGCTCTAGGAGCGGCAGCCCGTAGGCGCGGAACTCGGACCGCTTGTCAAGCCGACCGCGCTCGGGGACTACGAGAAGCAGGTCTAGTAGGACTAGCTGTTCTGGCCGAAGGTAGGCTC

1330 1340 1350 1360 1370 1380 1390 1400 1410 1420 1430 1440

```
TACGTGCTCGCTCGATGCGATGTTTCGCTTGGTGGTCGAATGGGCAGGTAGCCGGATCAAGCGTATGCAGCCGCCGCATTGCATCAGCCATGATGGATACTTTCTCGGCAGGAGCAAGGT
ATGCACGAGCGAGCTACGCTACAAAGCGAACCACCAGCTTACCCGTCCATCGGCCTAGTTCGCATACGTCGGCGGCGTAACGTAGTCGGTACTACCTATGAAAGAGCCGTCCTCGTTCCA
10 20 30 40 50 60 70 80 90 100 110 120

GAGATGACAGGAGATCCTGCCCCGGCACTTCGCCCAATAGCAGCCAGTCCCTTCCCGCTTCAGTGACAACGTCGAGCACAGCTGCGCAAGGAACGCCCGTCGTGGCCAGCCACGATAGCC
CTCTACTGTCCTCTAGGACGGGGCCGTGAAGCGGGTTATCGTCGGTCAGGGAAGGGCGAAGTCACTGTTGCAGCTCGTGTCGACGCGTTCCTTGCGGGCAGCACCGGTCGGTGCTATCGG
130 140 150 160 170 180 190 200 210 220 230 240

GCGCTGCCTCGTCTTGGAGTTCATTCAGGGCACCGGACAGGTCGGTCTTGACAAAAAGAACCGGGCGCCCCTGCGCTGACAGCCGGAACACGGCGGCATCAGAGCAGCCGATTGTCTGTT
CGCGACGGAGCAGAACCTCAAGTAAGTCCCGTGGCCTGTCCAGCCAGAACTGTTTTTCTTGGCCCGCGGGGACGCGACTGTCGGCCTTGTGCCGCCGTAGTCTCGTCGGCTAACAGACAA
250 260 270 280 290 300 310 320 330 340 350 360

GTGCCCAGTCATAGCCGAATAGCCTCTCCACCCAAGCGGCCGGAGAACCTGCGTGCAATCCATCTTGTTCAATCATGCCTCGATCGAGTTGAGAGTGAATATGAGACTCTAATTGGATAC
CACGGGTCAGTATCGGCTTATCGGAGAGGTGGGTTCGCCGGCCTCTTGGACGCACGTTAGGTAGAACAAGTTAGTACGGAGCTAGCTCAACTCTCACTTATACTCTGAGATTAACCTATG
370 380 390 400 410 420 430 440 450 460 470 480

CGAGGGGAATTTATGGAACGTCAGTGGAGCATTTTTGACAAGAAATATTTGCTAGCTGATAGTGACCTTAGGCGACTTTTGAACGCGCAATAATGGTTTCTGACGTATGTGCTTAGCTCA
GCTCCCCTTAAATACCTTGCAGTCACCTCGTAAAAACTGTTCTTTATAAACGATCGACTATCACTGGAATCCGCTGAAAACTTGCGCGTTATTACCAAAGACTGCATACACGAATCGAGT
490 500 510 520 530 540 550 560 570 580 590 600

TTAAACTCCAGAAACCCGCGGCTGAGTGGCTCCTTCAACGTTGCGGTTCTGTCAGTTCCAAACGTAAAACGGCTTGTCCCGCGTCATCGGCGGGGGTCATAACGTGACTCCCTTAATTCT
AATTTGAGGTCTTTGGGCGCCGACTCACCGAGGAAGTTGCAACGCCAAGACAGTCAAGGTTTGCATTTTGCCGAACAGGGCGCAGTAGCCGCCCCCAGTATTGCACTGAGGGAATTAAGA
610 620 630 640 650 660 670 680 690 700 710 720

CATGTATGATAATTCGAGGGTACCCGGGGATCCTCTAGAGGGCCCGACGTCGCATGCCTGCAGGTCACTGGATTTTGGTTTTAGGAATTAGAAATTTTATTGATAGAAGTATTTTACAAA
GTACATACTATTAAGCTCCCATGGGCCCCTAGGAGATCTCCCGGGCTGCAGCGTACGGACGTCCAGTGACCTAAAACCAAAATCCTTAATCTTTAAAATAACTATCTTCATAAAATGTTT
T
730 740 750 760 770 780 790 800 810 820 830 840

TACAAATACATACTAAGGGTTTCTTATATGCTCAACACATGAGCGAAACCCTATAAGAACCCTAATTCCCTTATCTGGGAACTACTCACACATTATTCTGGAGAAAAATAGAGAGAGATA
ATGTTTATGTATGATTCCCAAAGAATATACGAGTTGTGTACTCGCTTTGGGATATTCTTGGGATTAAGGGAATAGACCCTTGATGAGTGTGTAATAAGACCTCTTTTTATCTCTCTCTAT
850 860 870 880 890 900 910 920 930 940 950 960

GATTTGTAGAGAGAGACTGGTGATTTTTGCGGACTCTAGCATGGCCGCGGGATATCACCACTTTGTACAAGAAAGCTGGGTCGGCGCGCCCACCCTTGCCGGCATCGATCAATTGGAGCT
CTAAACATCTCTCTCTGACCACTAAAAACGCCTGAGATCGTACCGGCGCCCTATAGTGGTGAAACATGTTCTTTCGACCCAGCCGCGCGGGTGGGAACGGCCGTAGCTAGTTAACCTCGA
attB2
970 980 990 1000 1010 1020 1030 1040 1050 1060 1070 1080

CTCCCATATGATGGGTAAGAATTACAAGTCCCTGGACTCTGTTGTGGCCTCTGACTTCATAGCCCTAGGTATCACCTCTGAAGTTGCTGAGACACTCCATGGTAGACTGGCCGAGATCGT
GAGGGTATACTACCCATTCTTAATGTTCAGGGACCTGAGACAACACCGGAGACTGAAGTATCGGGATCCATAGTGGAGACTTCAACGACTCTGTGAGGTACCATCTGACCGGCTCTAGCA
H T L I V L G Q V R N H G R V E Y G * T D G R F N S L C E M T S Q G L D H
AAE
1090 1100 1110 1120 1130 1140 1150 1160 1170 1180 1190 1200

GTGTAATTATGGCGCTGCCACTCCCCAAACATGGATCAATATTGCCAACCATATTCTGTCGCCTGACCTCCCCTTCTCCCTGCACCAGATGCTCTTCTATGGTTGCTATAAAGACTTTGG
CACATTAATACCGCGACGGTGAGGGGTTTGTACCTAGTTATAACGGTTGGTATAAGACAGCGGACTGGAGGGGAAGAGGGACGTGGTCTACGAGAAGATACCAACGATATTTCTGAAACC
T I I A S G S G L C P D I N G V M N Q R R V E G E G Q V L H E E I T A I F V K S
1210 1220 1230 1240 1250 1260 1270 1280 1290 1300 1310 1320
```

```
ACCTGCCCCTCCTGCTTGGATACCCGACCCGGAGAAAGTAAAGTCCACCAATCTGGGCGCACTTTTGGAGAAGCGAGGAAAAGAGTTTTTGGGAGTCAAGTATAAGGATCCCATTTCAAG
TGGACGGGGAGGACGAACCTATGGGCTGGGCCTCTTTCATTTCAGGTGGTTAGACCCGCGTGAAAACCTCTTCGCTCCTTTTCTCAAAAACCCTCAGTTCATATTCCTAGGGTAAAGTTC
 R  G  R  R  S  P  Y  G  V  R  L  F  Y  L  G  G  I  Q  A  C  K  Q  L  L  S  S  F  L  K  Q  S  D  L  I  L  I  G  N  *  A
10 20 30 40 50 60 70 80 90 100 110 120

CTTTTCTCATTTCCAAGAATTTTCTGTAAGAAACCCTGAGGTGTATTGGAGAACAGTACTAATGGATGAGATGAAGATAAGTTTTTCAAAGGATCCAGAATGTATATTGCGTAGAGATGA
GAAAAGAGTAAAGGTTCTTAAAAGACATTCTTTGGGACTCCACATAACCTCTTGTCATGATTACCTACTCTACTTCTATTCAAAAAGTTTCCTAGGTCTTACATATAACGCATCTCTACT
 K  R  M  E  L  F  K  R  Y  S  V  R  L  H  I  P  S  C  Y  *  H  I  L  H  L  Y  T  K  *  L  I  W  F  T  Y  Q  T  S  I  I
130 140 150 160 170 180 190 200 210 220 230 240

TATTAATAATCCAGGGGGTAGTGAATGGCTTCCAGGAGGTTATCTTAACTCAGCAAAGAATTGCTTGAATGTAAATAGTAACAAGAAATTGAATGATACAATGATTGTATGGCGTGATGA
ATAATTATTAGGTCCCCCATCACTTACCGAAGGTCCTCCAATAGAATTGAGTCGTTTCTTAACGAACTTACATTTATCATTGTTCTTTAACTTACTATGTTACTAACATACCGCACTACT
 N  I  I  W  P  T  T  F  P  K  W  S  T  I  K  V  *  C  L  I  A  Q  I  Y  I  T  V  L  F  Q  I  I  C  H  N  Y  P  T  I  F
250 260 270 280 290 300 310 320 330 340 350 360

AGGAAATGATGATTTGCCTCTAAACAAATTGACACTTGACCAATTGCGTAAACGTGTTTGGTTAGTTGGTTATGCACTTGAAGAAATGGGTTTGGAGAAGGGTTGTGCAATTGCAATTGA
TCCTTTACTACTAAACGGAGATTTGTTTAACTGTGAACTGGTTAACGCATTTGCACAAACCAATCAACCAATACGTGAACTTCTTTACCCAAACCTCTTCCCAACACGTTAACGTTAACT
 S  I  I  I  Q  R  *  V  F  Q  C  K  V  L  Q  T  F  T  N  P  *  N  T  I  C  K  F  F  H  T  Q  L  L  T  T  C  N  C  N  I
370 380 390 400 410 420 430 440 450 460 470 480

TATGCCAATGCATGTGGATGCTGTGGTTATCTATCTAGCTATTGTTCTTGCGGGATATGTAGTTGTTTCTATTGCTGATAGTTTTTCTGCTCCTGAAATATCAACAAGACTTCGACTATC
ATACGGTTACGTACACCTACGACACCAATAGATAGATCGATAACAAGAACGCCCTATACATCAACAAAGATAACGACTATCAAAAAGACGAGGACTTTATAGTTGTTCTGAAGCTGATAG
 H  W  H  M  H  I  S  H  N  D  I  *  S  N  N  K  R  S  I  Y  N  N  R  N  S  I  T  K  R  S  R  F  Y  *  C  S  K  S  *  *
490 500 510 520 530 540 550 560 570 580 590 600

AAAAGCAAAAGCCATTTTTACACAGGATCATATTATTCGTGGGAAGAAGCGTATTCCCTTATACAGTAGAGTTGTGGAAGCCAAGTCTCCCATGGCCATTGTTATTCCTTGTAGTGGCTC
TTTTCGTTTTCGGTAAAAATGTGTCCTAGTATAATAAGCACCCTTCTTCGCATAAGGGAATATGTCATCTCAACACCTTCGGTTCAGAGGGTACCGGTAACAATAAGGAACATCACCGAG
 F  C  F  G  N  K  C  L  I  M  N  N  T  P  L  L  T  N  G  *  V  T  S  N  H  F  G  L  R  G  H  G  N  N  N  R  T  T  A  R
610 620 630 640 650 660 670 680 690 700 710 720

TAATATTGGTGCAGAATTGCGTGATGGCGATATTTCTTGGGATTACTTTCTAGAAAGAGCAAAAGAGTTTAAAAATTGTGAATTTACTGCTAGAGAACAACCAGTTGATGCCTATACAAA
ATTATAACCACGTCTTAACGCACTACCGCTATAAAGAACCCTAATGAAAGATCTTTCTCGTTTTCTCAAATTTTTAACACTTAAATGACGATCTCTTGTTGGTCAACTACGGATATGTTT
 I  N  T  C  F  Q  T  I  A  I  N  R  P  I  V  K  *  F  S  C  F  L  K  F  I  T  F  K  S  S  S  F  L  W  N  I  G  I  C  V
730 740 750 760 770 780 790 800 810 820 830 840

CATCCTCTTCTCATCTGGAACAACAGGGGAGCCAAAGGCAATTCCATGGACTCAAGCAACTCCTTTAAAAGCAGCTGCAGATGGGTGGAGCCATTTGGACATTAGGAAAGGTGATGTCAT
GTAGGAGAAGAGTAGACCTTGTTGTCCCCTCGGTTTCCGTTAAGGTACCTGAGTTCGTTGAGGAAATTTTCGTCGACGTCTACCCACCTCGGTAAACCTGTAATCCTTTCCACTACAGTA
 D  E  E  *  R  S  C  C  P  L  W  L  C  N  W  P  S  L  C  S  R  *  F  C  S  C  I  P  P  A  M  Q  V  N  P  F  T  I  D  N
850 860 870 880 890 900 910 920 930 940 950 960

TGTTTGGCCCACTAATCTTGGTTGGATGATGGGTCCTTGGCTGGTCTATGCTTCACTCCTTAATGGGGCTTCTATTGCCTTGTATAATGGATCACCACTTGTTTCTGGCTTTGCCAAATT
ACAAACCGGGTGATTAGAACCAACCTACTACCCAGGAACCGACCAGATACGAAGTGAGGAATTACCCCGAAGATAACGGAACATATTACCTAGTGGTGAACAAAGACCGAAACGGTTTAA
 N  P  G  S  I  K  T  P  H  H  T  R  P  Q  D  I  S  *  E  K  I  P  S  R  N  G  Q  I  I  S  *  W  K  N  R  A  K  G  F  K
970 980 990 1000 1010 1020 1030 1040 1050 1060 1070 1080

TGTGCAGGATGCTAAAGTAACAATGCTAGGTGTGGTCCCTAGTATTGTTCGATCATGGAAAAGTACCAATTGTGTTAGTGGCTATGATTGGTCCACCATCCGTTGCTTTTCCTCTTCTGG
ACACGTCCTACGATTTCATTGTTACGATCCACACCAGGGATCATAACAAGCTAGTACCTTTTCATGGTTAACACAATCACCGATACTAACCAGGTGGTAGGCAACGAAAAGGAGAAGACC
 H  L  I  S  F  Y  C  H  *  T  H  D  R  T  N  N  S  *  P  F  T  G  I  T  N  T  A  I  I  P  G  G  D  T  A  K  G  R  R  T
1090 1100 1110 1120 1130 1140 1150 1160 1170 1180 1190 1200
```

```
TGAAGCATCTAATGTAGATGAATACCTATGGTTGATGGGGAGAGCAAACTACAAGCCTGTTATCGAAATGTGTGGTGGCACAGAAATTGGTGGTGCATTTTCTGCTGGCTCTTTCTTACA
ACTTCGTAGATTACATCTACTTATGGATACCAACTACCCCTCTCGTTTGATGTTCGGACAATAGCTTTACACACCACCGTGTCTTTAACCACCACGTAAAAGACGACCGAGAAAGAATGT
 F  C  R  I  Y  I  F  V  *  P  Q  H  P  S  C  V  V  L  R  N  D  F  H  T  T  A  C  F  N  T  T  C  K  R  S  A  R  E  *  L
10 20 30 40 50 60 70 80 90 100 110 120
```

```
AGCTCAATCATTATCTTCATTTAGTTCACAATGTATGGGTTGCACTTTATACATACTTGACAAGAATGGTTATCCAATGCCTAAAAACAAACCAGGAATTGGTGAATTAGCGCTTGGTCC
TCGAGTTAGTAATAGAAGTAAATCAAGTGTTACATACCCAACGTGAAATATGTATGAACTGTTCTTACCAATAGGTTACGGATTTTTGTTTGGTCCTTAACCACTTAATCGCGAACCAGG
 S  L  *  *  R  *  K  T  *  L  T  H  T  A  S  *  V  Y  K  V  L  I  T  I  W  H  R  F  V  F  W  S  N  T  F  *  R  K  T  W
130 140 150 160 170 180 190 200 210 220 230 240
```

```
AGTCATGTTTGGAGCATCGAAGACTCTGTTGAATGGTAATCACCATGATGTTTATTTTAAGGGAATGCCTACATTGAATGGAGAGGTTTTAAGGAGGCATGGGGACATTTTTGAGCTTAC
TCAGTACAAACCTCGTAGCTTCTGAGACAACTTACCATTAGTGGTACTACAAATAAAATTCCCTTACGGATGTAACTTACCTCTCCAAAATTCCTCCGTACCCCTGTAAAAACTCGAATG
 D  H  K  S  C  R  L  S  Q  Q  I  T  I  V  M  I  N  I  K  L  S  H  R  C  Q  I  S  L  N  *  P  P  M  P  V  N  K  L  K  C
250 260 270 280 290 300 310 320 330 340 350 360
```

```
ATCTAATGGTTATTATCATGCACATGGTCGTGCAGATGATACAATGAATATTGGAGGCATCAAGATTAGTTCCATAGAGATTGAACGAGTTTGTAATGAAGTTGATGACAGAGTTTTCGA
TAGATTACCAATAATAGTACGTGTACCAGCACGTCTACTATGTTACTTATAACCTCCGTAGTTCTAATCAAGGTATCTCTAACTTGCTCAAACATTACTTCAACTACTGTCTCAAAAGCT
 R  I  T  I  I  M  C  M  T  T  C  I  I  C  H  I  N  S  A  D  L  N  T  G  Y  L  N  F  S  N  T  I  F  N  I  V  S  N  E  L
370 380 390 400 410 420 430 440 450 460 470 480
```

```
GACAACTGCTATTGGAGTGCCACCTTTGGGCGGTGGACCTGAGCAATTAGTAATTTTCTTTGTATTAAAAGATTCAAATGATACAACTATTGACTTAAATCAATTGAGGTTATCTTTCAA
CTGTTGACGATAACCTCACGGTGGAAACCCGCCACCTGGACTCGTTAATCATTAAAAGAAACATAATTTTCTAAGTTTACTATGTTGATAACTGAATTTAGTTAACTCCAATAGAAAGTT
 C  S  S  N  S  H  W  R  Q  A  T  S  R  L  L  *  Y  N  E  K  Y  *  F  I  *  I  I  C  S  N  V  *  I  L  Q  P  *  R  E  V
490 500 510 520 530 540 550 560 570 580 590 600
```

```
CTTGGGTTTACAGAAGAAACTAAATCCTCTGTTCAAGGTCACTCGTGTTGTGCCTCTTTCATCACTTCCGAGAACAGCAACCAACAAGATCATGAGAAGGGTTTTGCGCCAGCAATTTTC
GAACCCAAATGTCTTCTTTGATTTAGGAGACAAGTTCCAGTGAGCACAACACGGAGAAAGTAGTGAAGGCTCTTGTCGTTGGTTGTTCTAGTACTCTTCCCAAAACGCGGTCGTTAAAAG
 Q  T  *  L  L  F  *  I  R  Q  E  L  D  S  T  N  H  R  K  *  *  K  R  S  C  C  G  V  L  D  H  S  P  N  Q  A  L  L  K  R
610 620 630 640 650 660 670 680 690 700 710 720
```

```
TCACTTTGAATGAGTCGACCTGCAGGCGGCCGCGAATTCACTAGTGATTCTAGACTTTACCCTAACTACTCCTTTCTCAGTTGGCTAGCTTCAAAAAACAGAGTTGCCACGGGAGACAGA
AGTGAAACTTACTCAGCTGGACGTCCGCCGGCGCTTAAGTGATCACTAAGATCTGAAATGGGATTGATGAGGAAAGAGTCAACCGATCGAAGTTTTTTGTCTCAACGGTGCCCTCTGTCT
 V  K  F  S
pAtRBCS2B
730 740 750 760 770 780 790 800 810 820 830 840
```

```
ATGTTTAAATGGAATCAACAAGTGAACACAAACTGCATGTTCTGTCACGAACCTATGGAAACAAGACACCATCTTTTTTTTGGGTGCTCTTACTCGCAGAAGGTATGGGAGAAGCTGATA
TACAAATTTACCTTAGTTGTTCACTTGTGTTTGACGTACAAGACAGTGCTTGGATACCTTTGTTCTGTGGTAGAAAAAAAACCCACGAGAATGAGCGTCTTCCATACCCTCTTCGACTAT
850 860 870 880 890 900 910 920 930 940 950 960
```

```
AGGGGAATACTACTTGATAAGTACTCGACATATTGGAGGGAAGTCTTTAAAACCATTTGTGACAAAAACTATGACAAAACAAAAATCTTCATTCTACGGTATGTCTTCCAAAACACGGTT
TCCCCTTATGATGAACTATTCATGAGCTGTATAACCTCCCTTCAGAAATTTGGTAAACACTGTTTTTGATACTGTTTTGTTTTTAGAAGTAAGATGCCATACAGAAGGTTTTGTGCCAA
970 980 990 1000 1010 1020 1030 1040 1050 1060 1070 1080
```

```
CATTCGATTTGGGGAGAAAGAAATGCTTGAAAGCATGGTGAACAACCATCACCAATGGAGAAACTTGTCAAGTTAATTGACAAAAATGTTTGGAACCGGCTGAGCACAATACGGAGTGGG
GTAAGCTAAACCCCTCTTTCTTTACGAACTTTCGTACCACTTGTTGGTAGTGGTTACCTCTTTGAACAGTTCAATTAACTGTTTTTACAAACCTTGGCCGACTCGTGTTATGCCTCACCC
1090 1100 1110 1120 1130 1140 1150 1160 1170 1180 1190 1200
```

GGGAGCTTTGAAGTATGAAGGAGGTCTTCAAGTTTGGTTTGGTACAAGACAAGCTTAAGACCAAATCCTCTGTTTTAGATTTGTTTTTCAAAAAAGTATGCATTAAGTTGTAAACACGTT
CCCTCGAAACTTCATACTTCCTCCAGAAGTTCAAACCAAACCATGTTCTGTTCGAATTCTGGTTTAGGAGACAAAATCTAAACAAAAAGTTTTTTCATACGTAATTCAACATTTGTGCAA

10 20 30 40 50 60 70 80 90 100 110 120

TATTTCTCTTTTTAAAATCTAATCTAAAAATTTTCTTTTTTAAAATGACATAGAAAAATCCAACTGTATATTTTAAATCCATAAACATGAATTCTAGAGCCGCAAGTCCAAAAATTTAGA
ATAAAGAGAAAAATTTTAGATTAGATTTTTAAAAGAAAAAATTTTACTGTATCTTTTTAGGTTGACATATAAAATTTAGGTATTTGTACTTAAGATCTCGGCGTTCAGGTTTTTAAATCT

130 140 150 160 170 180 190 200 210 220 230 240

GATGACATCATAGCAAGCAAGGACACGCAAGAATTAATGAAATTTATAATGGAGATAACAAAGCATGAATATTTATAATTTGGAAACTGTTTTACATTAAATTAGATAGTTTACGATACA
CTACTGTAGTATCGTTCGTTCCTGTGCGTTCTTAATTACTTTAAATATTACCTCTATTGTTTCGTACTTATAAATATTAAACCTTTGACAAAATGTAATTTAATCTATCAAATGCTATGT

250 260 270 280 290 300 310 320 330 340 350 360

TTCAAAGCTAAAACACTTATTTACAATGAAAAGTAATCAATGTTTCTTGAATGGGTTTGGCTACTTCTTCTCATATGGTTGGACCGGGGCATATATGAGCTTTTACGTGGTATTCGTGGA
AAGTTTCGATTTTGTGAATAAATGTTACTTTTCATTAGTTACAAAGAACTTACCCAAACCGATGAAGAAGAGTATACCAACCTGGCCCCGTATATACTCGAAAATGCACCATAAGCACCT

370 380 390 400 410 420 430 440 450 460 470 480

CCCATGACCATATGAGCTTCTTCATGTGGCCTTAGAGTGTGGATTCTTCCCTCGGATCCACACCATCCATTGAAGTGTGTTATAAAATTTCCCCATGATCCGTCATGGCTTTTGTGCTTA
GGGTACTGGTATACTCGAAGAAGTACACCGGAATCTCACACCTAAGAAGGGAGCCTAGGTGTGGTAGGTAACTTCACACAATATTTTAAAGGGGTACTAGGCAGTACCGAAAACACGAAT

490 500 510 520 530 540 550 560 570 580 590 600

AGTTGTCATGGTGGGAATTATCTTGAGGTGCCACATTCGTGCTTAAGTGGGCCGTAAGTGCAATTGCGTTGTGCTCCAAAAGGAACGGTGCCGTGGGTTCCTCTTGTTCATCAGAAATAT
TCAACAGTACCACCCTTAATAGAACTCCACGGTGTAAGCACGAATTCACCCGGCATTCACGTTAACGCAACACGAGGTTTTCCTTGCCACGGCACCCAAGGAGAACAAGTAGTCTTTATA

610 620 630 640 650 660 670 680 690 700 710 720

ATTAATTAGCCGTAAAACCTGAAAATTCACAAGCATTTGGATTGTTTTCTAATTTAATATCCATTATGTGACTAAAAGTTCTAGTGATCGTACATACTACATAGAAAATAATAACACAAA
TAATTAATCGGCATTTTGGACTTTTAAGTGTTCGTAAACCTAACAAAAGATTAAATTATAGGTAATACACTGATTTTCAAGATCACTAGCATGTATGATGTATCTTTTATTATTGTGTTT

730 740 750 760 770 780 790 800 810 820 830 840

ATACTAGTTTACATTTCCCAATTAAAAACCATTTTGAATGAACTCTGTCTGATTTAATTATACTTTTAAAATGTGGGATGAATTCAAAGATTATACTTATATTCTTATTATTTAAGATTA
TATGATCAAATGTAAAGGGTTAATTTTTGGTAAAACTTACTTGAGACAGACTAAATTAATATGAAAATTTTACACCCTACTTAAGTTTCTAATATGAATATAAGAATAATAAATTCTAAT

850 860 870 880 890 900 910 920 930 940 950 960

TCAAGTGGAAAAATAAAAATATGAATGTGTTAATATAAGGTAATAGAAATTTAATCATTTTTTTAATCTATATGTAAAAAGTATTTAACCGATATCTACAATTTGACGCCTCCCAATTGA
AGTTCACCTTTTTATTTTTATACTTACACAATTATATTCCATTATCTTTAAATTAGTAAAAAAATTAGATATACATTTTTCATAAATTGGCTATAGATGTTAAACTGCGGAGGGTTAACT

970 980 990 1000 1010 1020 1030 1040 1050 1060 1070 1080

AAGGAGCCAAAAGCAACCGATCAAGTGGAGACCAGTAGCCATACACATTCACTCCTACCCTTACATGAGAAAGATAAGATTATGGAGTTTTCTGCCACGTGATCTTATCCTAGTGGTCCA
TTCCTCGGTTTTCGTTGGCTAGTTCACCTCTGGTCATCGGTATGTGTAAGTGAGGATGGGAATGTACTCTTTCTATTCTAATACCTCAAAAGACGGTGCACTAGAATAGGATCACCAGGT

1090 1100 1110 1120 1130 1140 1150 1160 1170 1180 1190 1200

AATCGATAAGGGTGTCAACACCTTTCCTTAATCCTGTGGCAATTAACGACGTTATCATGAATTATGGCCCCTTTGATCATTAGGGCTAGTTGCCTCTAGCGGTTCCCACTATATAAAGAT
TTAGCTATTCCCACAGTTGTGGAAAGGAATTAGGACACCGTTAATTGCTGCAATAGTACTTAATACCGGGGAAACTAGTAATCCCGATCAACGGAGATCGCCAAGGGTGATATATTTCTA

1210 1220 1230 1240 1250 1260 1270 1280 1290 1300 1310 1320

```
GACAAAACCAACAGACAAACAAGTAAGTAAGAGAAAAACCAAAAGAAGAAGAGAAACAACAAGAAGAAGTCTCGAGCCATGGTGCAAACACACAAGACAATGGACAATGGTACCACATCA
CTGTTTTGGTTGTCTGTTTGTTCATTCATTCTCTTTTTGGTTTTCTTCTTCTCTTTGTTGTTCTTCTTCAGAGCTCGGTACCACGTTTGTGTGTTCTGTTACCTGTTACCATGGTGTAGT
        10        20        30        40        50        60        70        80        90       100       110       120

ACAAAGCCAAGGAAGGGTACTTTCTTAATCTGAAGCTAAGAAGAACTTGAAGTTTGTTCAATGCAATGGGTAGTAGTGATTAAAAAAATGGTGTAGGTGTTTAAGATCTACTTACGTAAT
TGTTTCGGTTCCTTCCCATGAAAGAATTAGACTTCGATTCTTCTTGAACTTCAAACAAGTTACGTTACCCATCATCACTAATTTTTTTACCACATCCACAAATTCTAGATGAATGCATTA
       130       140       150       160       170       180       190       200       210       220       230       240

CAAGCTTGAATTCCGAATTCCCGATCTAGTAACATAGATGACACCGCGCGCGATAATTTATCCTAGTTTGCGCGCTATATTTTGTTTTCTATCGCGTATTAAATGTATAATTGCGGGACT
GTTCGAACTTAAGGCTTAAGGGCTAGATCATTGTATCTACTGTGGCGCGCGCTATTAAATAGGATCAAACGCGCGATATAAAACAAAAGATAGCGCATAATTTACATATTAACGCCCTGA
             T
       250       260       270       280       290       300       310       320       330       340       350       360

CTAATCATAAAAACCCATCTCATAAATAACGTCATGCATTACATGTTAATTATTACATGCTTAACGTAATTCAACAGAAATTATATGATAATCATCGCAAGACCGGCAACAGGATTCAAT
GATTAGTATTTTTGGGTAGAGTATTTATTGCAGTACGTAATGTACAATTAATAATGTACGAATTGCATTAAGTTGTCTTTAATATACTATTAGTAGCGTTCTGGCCGTTGTCCTAAGTTA
       370       380       390       400       410       420       430       440       450       460       470       480

CTTAAGAAACTTTATTGCCAAATGTTTGAACGATCGGGGAAATTCGAGCTCGGATCCAATCGAATTCCCGCGGCCGCCATGGCGGCCGGGAGCATGCGACGTCGGCGCCTTAATATTTGA
GAATTCTTTGAAATAACGGTTTACAAACTTGCTAGCCCCTTTAAGCTCGAGCCTAGGTTAGCTTAAGGGCGCCGGCGGTACCGCCGGCCCTCGTACGCTGCAGCCGCGGAATTATAAACT
                                                                                                                 *  Y  K  I
                                                                                                                 OLS
       490       500       510       520       530       540       550       560       570       580       590       600

TGGGAACACTACGCACGACCACTCTTTCGACAGTCAAACCTGGTCCAAACCCAAAAAGAACACCCCACTCAAATCCATCTCCAGTGGTAGACTTCCCTTCCTCCAACGACCTCTTCCTCA
ACCCTTGTGATGCGTGCTGGTGAGAAAGCTGTCAGTTTGGACCAGGTTTGGGTTTTTCTTGTGGGGTGAGTTTAGGTAGAGGTCACCATCTGAAGGGAAGGAGGTTGCTGGAGAAGGAGT
  P  V  S  R  V  V  V  R  E  V  T  L  G  P  G  F  G  F  L  V  G  W  E  F  G  D  G  T  T  S  K  G  E  E  L  S  R  K  R  L
       610       620       630       640       650       660       670       680       690       700       710       720

ACTCATCCATAACAAACAAGACAGTTGAGCTAGACATATTCCCATGCTCACTCAGCACATGACGTGAATCCACAAACTTATCACTCTTTAGATGCAACTTCTCCTCCACTTTGTCCAAAA
TGAGTAGGTATTGTTTGTTCTGTCAACTCGATCTGTATAAGGGTACGAGTGAGTCGTGTACTGCACTTAGGTGTTTGAATAGTGAGAAATCTACGTTGAAGAGGAGGTGAAACAGGTTTT
  E  D  M  V  F  L  V  T  S  S  S  M  N  G  H  E  S  L  V  H  R  S  D  V  F  K  D  S  K  L  H  L  K  E  E  V  K  D  L  I
       730       740       750       760       770       780       790       800       810       820       830       840

TAGCTTTCCCACCTGGGTGTGTAATCCAAAATATGGAGTTCCAATCACTAATCCCAATAGGAGTAAATGCCTCAATCAAACATTTCTCAATATTATTAGAGATCAACATAGGCACATCCT
ATCGAAAGGGTGGACCCACACATTAGGTTTTATACCTCAAGGTTAGTGATTAGGGTTATCCTCATTTACGGAGTTAGTTTGTAAAGAGTTATAATAATCTCTAGTTGTATCCGTGTAGGA
  A  K  G  G  P  H  T  I  W  F  I  S  N  W  D  S  I  G  I  P  T  F  A  E  I  L  C  K  E  I  N  N  S  I  L  M  P  V  D  K
       850       860       870       880       890       900       910       920       930       940       950       960

TATGTAAATCAAATATCAGTCCTGCTTCCCTTATATGTCCCCCAATAGTTCCTTCCGAGTTTGGTAAGATTGTTTGCCCAGTTGACACCAACTCAAATATCGGCCTTTCCCCAACTGACT
ATACATTTAGTTTATAGTCAGGACGAAGGGAATATACAGGGGGTTATCAAGGAAGGCTCAAACCATTCTAACAAACGGGTCAACTGTGGTTGAGTTTATAGCCGGAAAGGGGTTGACTGA
  H  L  D  F  I  L  G  A  E  R  I  H  G  G  I  T  G  E  S  N  P  L  I  T  Q  G  T  S  V  L  E  F  I  P  R  E  G  V  S  E
       970       980       990      1000      1010      1020      1030      1040      1050      1060      1070      1080

CATCGGGTTCAGCTCCAACAATCACCGCAGCAGCCCCATCACCAAAGATAGCTTGTCCCACTAGTAATTCGAGGTCAGACTCTGAAGGCCCACGAAACAAGCAAGCCATTATGTCACAAC
GTAGCCCAAGTCGAGGTTGTTAGTGGCGTCGTCGGGGTAGTGGTTTCTATCGAACAGGGTGATCATTAAGCTCCAGTCTGAGACTTCCGGGTGCTTTGTTCGTTCGGTAATACAGTGTTG
  D  P  E  A  G  V  I  V  A  A  A  G  D  G  F  I  A  Q  G  V  L  L  E  L  D  S  E  S  P  G  R  F  L  C  A  M  I  D  C  C
      1090      1100      1110      1120      1130      1140      1150      1160      1170      1180      1190      1200
```

```
ACACGGCGAGAACTCGTGCGCCTTTGTTATTCTCTGCTATGTCCTTGGCAATGCGTAGAACGGTTCCACCACCATAACAGCCTAGTTGATACATCATCACACGCTTCACTGAGGGACTCA
TGTGCCGCTCTTGAGCACGCGGAAACAATAAGAGACGATACAGGAACCGTTACGCATCTTGCCAAGGTGGTGGTATTGTCGGATCAACTATGTAGTAGTGTGCGAAGTGACTCCCTGAGT
 V  A  L  V  R  A  G  K  N  N  E  A  I  D  K  A  I  R  L  V  T  G  G  G  Y  C  G  L  Q  Y  M  M  V  R  K  V  S  P  S  L
10 20 30 40 50 60 70 80 90 100 110 120

GTCCGAGAAGCTTAGCGCAATGGTAGTCTGCACCGGGCATGTCAGTGGTTGATGCGCTAGTGAAGATTAAATGAGTGATTTTAGACTTGGGTTGACCCCATTCTTTGATGGCCTTTGCAC
CAGGCTCTTCGAATCGCGTTACCATCAGACGTGGCCCGTACAGTCACCAACTACGCGATCACTTCTAATTTACTCACTAAAATCTGAACCCAACTGGGGTAAGAAACTACCGGAAACGTG
 G  L  L  K  A  C  H  Y  D  A  G  P  M  D  T  T  S  A  S  T  F  I  L  H  T  I  K  S  K  P  Q  G  W  E  K  I  A  K  A  C
130 140 150 160 170 180 190 200 210 220 230 240

AAGCATCCTTCCCAAGTTTTGGAACCTCAACTACCAACATGTCTTGACGTGCATCCAGAGTTTGCATCTCGTGCTCCACCAATCTTGGGTTTTGCTTTAGGTGTTCTTCATTTAAGAAAC
TTCGTAGGAAGGGTTCAAAACCTTGGAGTTGATGGTTGTACAGAACTGCACGTAGGTCTCAAACGTAGAGCACGAGGTGGTTAGAACCCAAAACGAAATCCACAAGAAGTAAATTCTTTG
 A  D  K  G  L  K  P  V  E  V  V  L  M  D  Q  R  A  D  L  T  Q  M  E  H  E  V  L  R  P  N  Q  K  L  H  E  E  N  L  F  C
250 260 270 280 290 300 310 320 330 340 350 360

AGTTACGTTTCCTTATCATACTTTTGTCACATATTTTTCGAAACTTTTCTTTGAGTTGAGTCATGTGTTCACTTTTGGTGACGCGAAAATAGTAGTCAGGAAACTCATCTTGTAATAAAA
TCAATGCAAAGGAATAGTATGAAAACAGTGTATAAAAAGCTTTGAAAAGAAACTCAACTCAGTACACAAGTGAAAACCACTGCGCTTTTATCATCAGTCCTTTGAGTAGAACATTATTTT
 N  R  K  R  I  M  S  K  D  C  I  K  R  F  K  E  K  L  Q  T  M  H  E  S  K  T  V  R  F  Y  Y  D  P  F  E  D  Q  L  L  I
370 380 390 400 410 420 430 440 450 460 470 480

TGTTCTCCGGATTGGCGGTGCCAATGGCGAGAACGGAGGCCGGACCCTCAGCACGAAGATGATTCATCCTAGGACCGGTGGTGAAGGGGGCGGCCGCGGAGCCTGCTTTTTTGTACAAAC
ACAAGAGGCCTAACCGCCACGGTTACCGCTCTTGCCTCCGGCCTGGGAGTCGTGCTTCTACTAAGTAGGATCCTGGCCACCACTTCCCCCGCCGGCGCCTCGGACGAAAAAACATGTTTG
 N  E  P  N  A  T  G  I  A  L  V  S  A  P  G  E  A  R  L  H  N  M
attB1
490 500 510 520 530 540 550 560 570 580 590 600

TTGTGATATCACTAGTGCGGCCGCCTGCAGGTCGACTAGAATAGTAAATTGTAATGTTGTTTGTTGTTTGTTTTGTTGTGGTATTGTTGTAAAAATACCGGAGTCCTCTCCAAATGAAAT
AACACTATAGTGATCACGCCGGCGGACGTCCAGCTGATCTTATCATTTAACATTACAACAAACAACAAACAAAACAACACCATAACAACATTTTTATGGCCTCAGGAGAGGTTTACTTTA
p35S
610 620 630 640 650 660 670 680 690 700 710 720

GAACTTCCTTATATAGAGGAAGGGTCTTGCGAAGGATAGTGGGATTGTGCGTCATCCCTTACGTCAGTGGAGATATCACATCAATCCACTTGCTTTGAAGACGTGGTTGGAACGTCTTCT
CTTGAAGGAATATATCTCCTTCCCAGAACGCTTCCTATCACCCTAACACGCAGTAGGGAATGCAGTCACCTCTATAGTGTAGTTAGGTGAACGAAACTTCTGCACCAACCTTGCAGAAGA
730 740 750 760 770 780 790 800 810 820 830 840

TTTTCCACGATGCTCCTCGTGGGTGGGGGTCCATCTTTGGGACCACTGTCGGCAGAGGCATCTTGAACGATAGCCTTTCCTTTATCGCAATGATGGCATTTGTAGGTGCCACCTTCCTTT
AAAAGGTGCTACGAGGAGCACCCACCCCCAGGTAGAAACCCTGGTGACAGCCGTCTCCGTAGAACTTGCTATCGGAAAGGAAATAGCGTTACTACCGTAAACATCCACGGTGGAAGGAAA
850 860 870 880 890 900 910 920 930 940 950 960

TCTACTGTCCTTTTGATGAAGTGACAGATAGCTGGGCAATGGAATCCGAGGAGGTTTCCCGATATTACCCTTTGTTGAAAAGTCTCAATAGCCCTTTGGTCTTCTGAGACTGTATCTTTG
AGATGACAGGAAAACTACTTCACTGTCTATCGACCCGTTACCTTAGGCTCCTCCAAAGGGCTATAATGGGAAACAACTTTTCAGAGTTATCGGGAAACCAGAAGACTCTGACATAGAAAC
970 980 990 1000 1010 1020 1030 1040 1050 1060 1070 1080

ATATTCTTGGAGTAGACGAGAGTGTCGTGCTCCACCATGTTGACGAAGATTTTCTTCTTGTCATTGAGTCGTAAAAGACTCTGTATGAACTGTTCGCCAGTCTTCACGGCGAGTTCTGTT
TATAAGAACCTCATCTGCTCTCACAGCACGAGGTGGTACAACTGCTTCTAAAAGAAGAACAGTAACTCAGCATTTTCTGAGACATACTTGACAAGCGGTCAGAAGTGCCGCTCAAGACAA
1090 1100 1110 1120 1130 1140 1150 1160 1170 1180 1190 1200

AGATCCTCGATCTGAATTTTTGACTCCATGGCCTTTGATTCAGTAGGAACTACTTTCTTAGAGACTCCAATCTCTATTACTTGCCTTGGTTTATGAAGCAAGCCTTGAATCGTCCATACT
TCTAGGAGCTAGACTTAAAAACTGAGGTACCGGAAACTAAGTCATCCTTGATGAAAGAATCTCTGAGGTTAGAGATAATGAACGGAACCAAATACTTCGTTCGGAACTTAGCAGGTATGA
1210 1220 1230 1240 1250 1260 1270 1280 1290 1300 1310 1320
```

GGAATAGTACTTCTGATCTTGAGAAATATATCTTTCTCTGTGTTCTTGATGCAGTTAGTCCTGAATCTTTTGACTGCATCTTTAACCTTCTTGGGAAGGTATTTGATCTCCTGGAGATTA
CCTTATCATGAAGACTAGAACTCTTTATATAGAAAGAGACACAAGAACTACGTCAATCAGGACTTAGAAAACTGACGTAGAAATTGGAAGAACCCTTCCATAAACTAGAGGACCTCTAAT

10 20 30 40 50 60 70 80 90 100 110 120

TTACTCGGGTAGATCGTCTTGATGAGACCTGCCGCGTAGGCCTCTCTAACCATCTGTGGGTCAGCATTCTTTCTGAAATTGAAGAGGCTAATCTTCTCATTATCGGTGGTGAACATGGTA
AATGAGCCCATCTAGCAGAACTACTCTGGACGGCGCATCCGGAGAGATTGGTAGACACCCAGTCGTAAGAAAGACTTTAACTTCTCCGATTAGAAGAGTAATAGCCACCACTTGTACCAT

130 140 150 160 170 180 190 200 210 220 230 240

TCGTCACCTTCTCCGTCGAACTTTCTTCCTAGATCGTAGAGATAGAGAAAGTCGTCCATGGTGATCTCCGGGGCAAAGGAGATCAGCTTGGCTCTAGTCGACCATATGGGAGAGCTCA
AGCAGTGGAAGAGGCAGCTTGAAAGAAGGATCTAGCATCTCTATCTCTTTCAGCAGGTACCACTAGAGGCCCCGTTTCCTCTAGTCGAACCGAGATCAGCTGGTATACCCTCTCGAGT

250 260 270 280 290 300 310 320 330 340 350 360

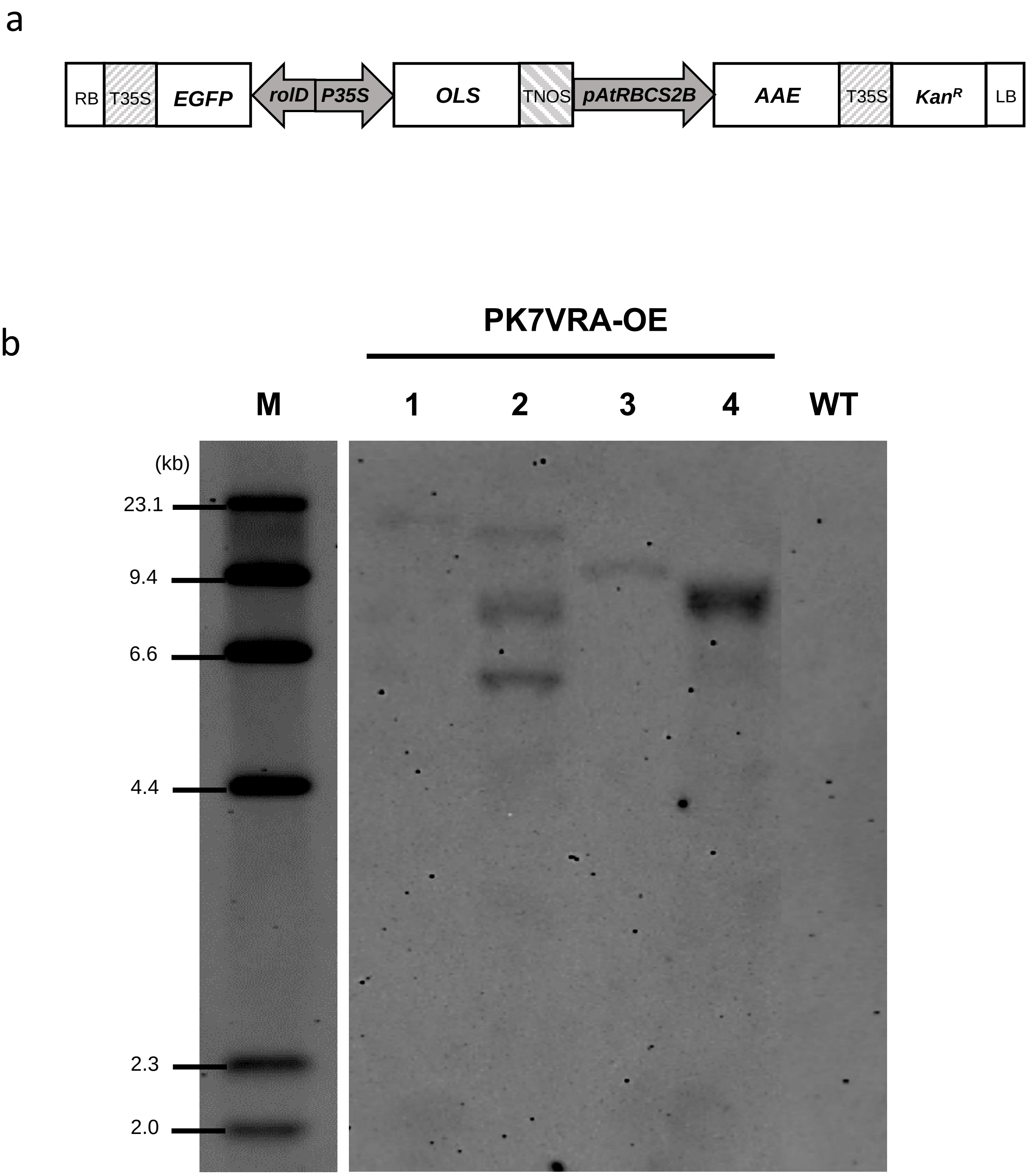

Figure S1. **Map of PK7VRA vector construction for the synthesis of cannabinoids and Southern blot image showing the number of transgene copies.** (a) Linear representation of the T-DNA of the PK7VRA construct. It is approximately 11kb from the right border (RB) to the Left Border (LB). Within the RB and LB, it consists of rol root loci D promoter (rolD); enhanced green fluorescent protein gene (EGFP); 35S terminator (T35S) and 35S promoter (P35S); OLS gene (1158bp); TNOS terminator (TNOS); light-inducible promoter (AtRBCS2B); AAE gene (2163 bp) and Kanamycin resistance gene (Kan^R). (b) gDNA from each line was digested with XhoI and probed with 35S promoter specific DIG-labelled probe. PK7VRA-OE; transgenic *N. benthamiana* lines overexpressing *acyl-activating enzyme* and *olivetol synthase*.

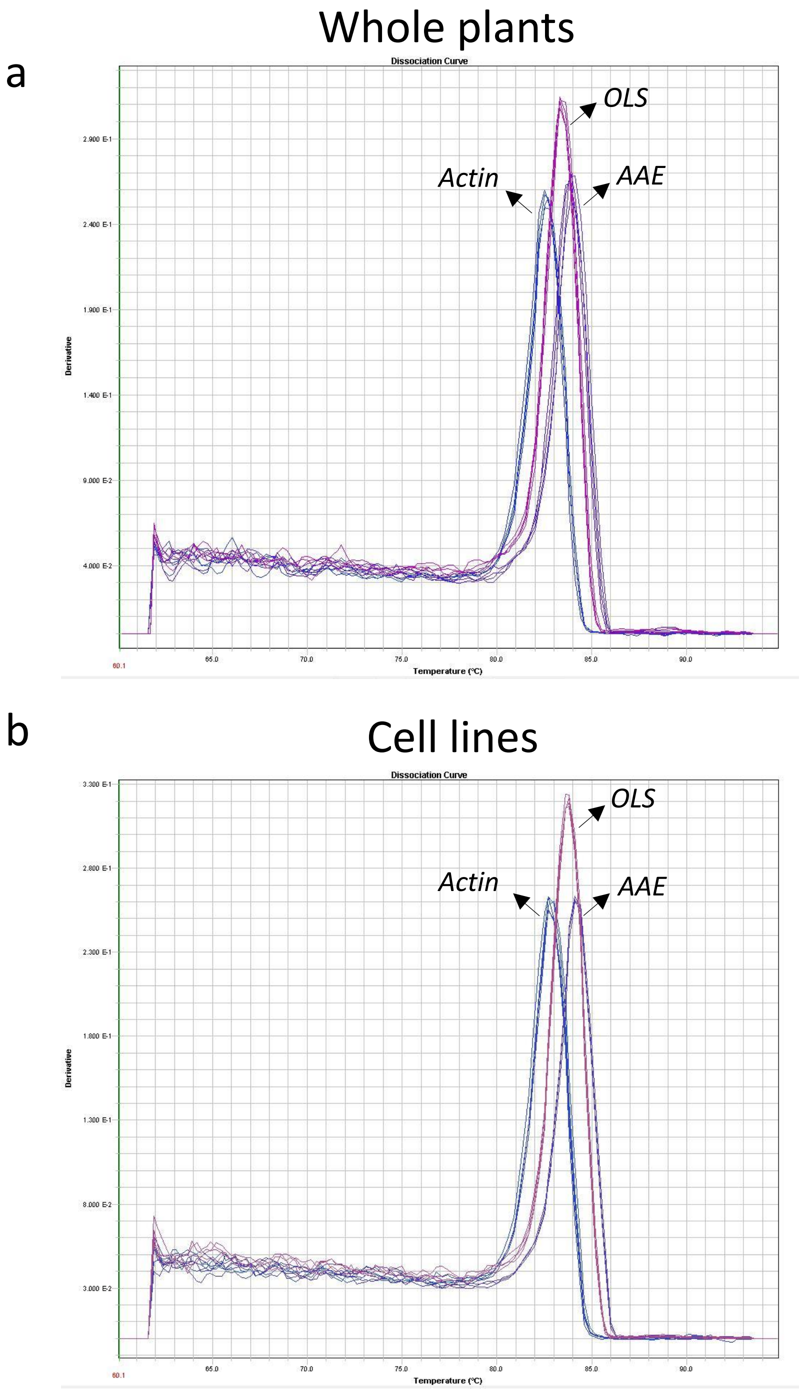

Figure S2. Dissociation curve analysis. (a) Dissociation curves of *OLS*, *AAE* and *Actin* genes shown in Figure 2d. (b) Dissociation curves of *OLS*, *AAE* and *Actin* genes shown in Figure 7d.

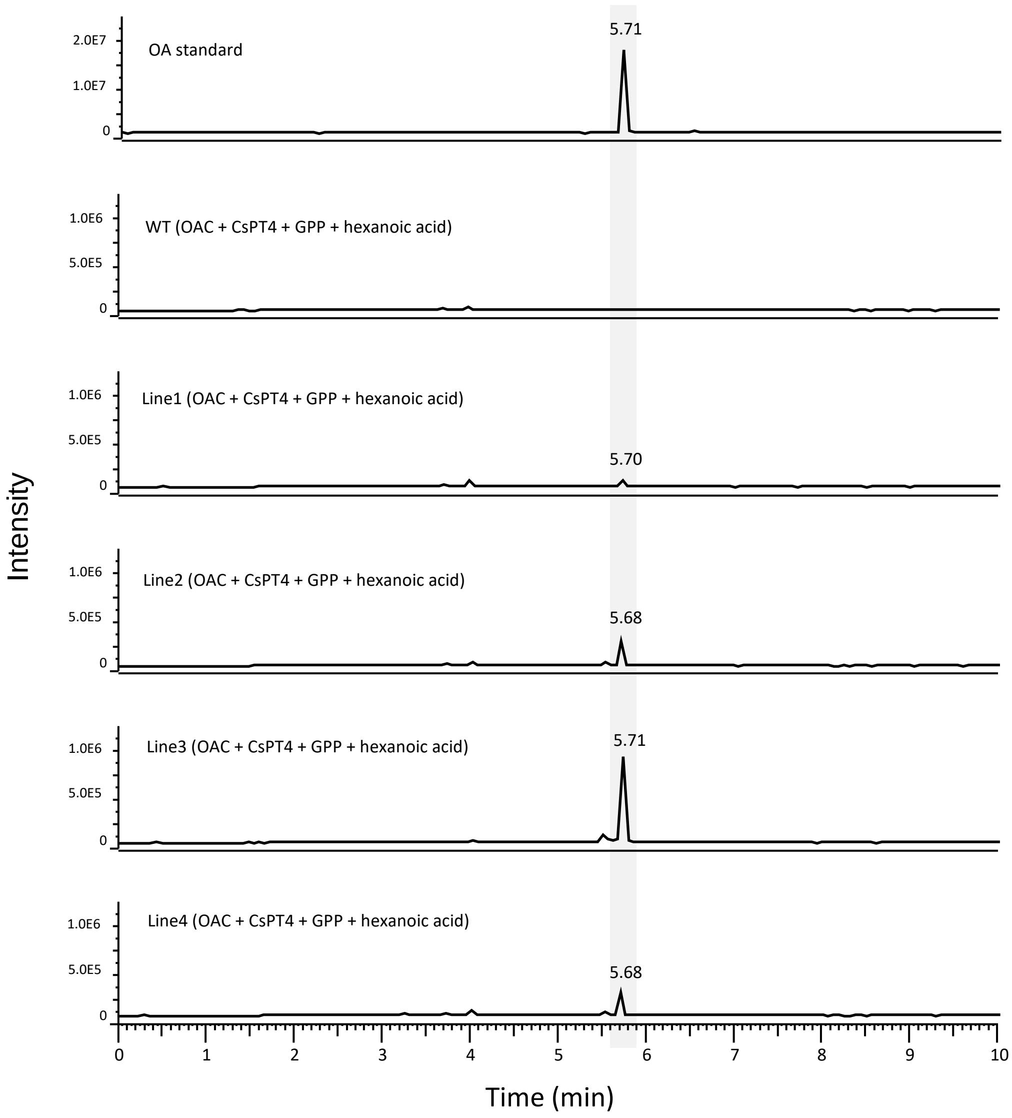

Figure S3. LC-MS results of transgenic *N. benthamiana* lines transiently expressing OAC and CsPT4. With the presence of hexanoic acid and GPP, transient expression of CsPT4 and OAC was able to generate olivetolic acid in PK7VRA plants. *OAC*; *olivetolic acid cyclase*, OA; olivetolic acid, GPP; geranyl diphosphate, *CsPT4; geranyldiphosphate:olivetolate geranyltransferase 4.*

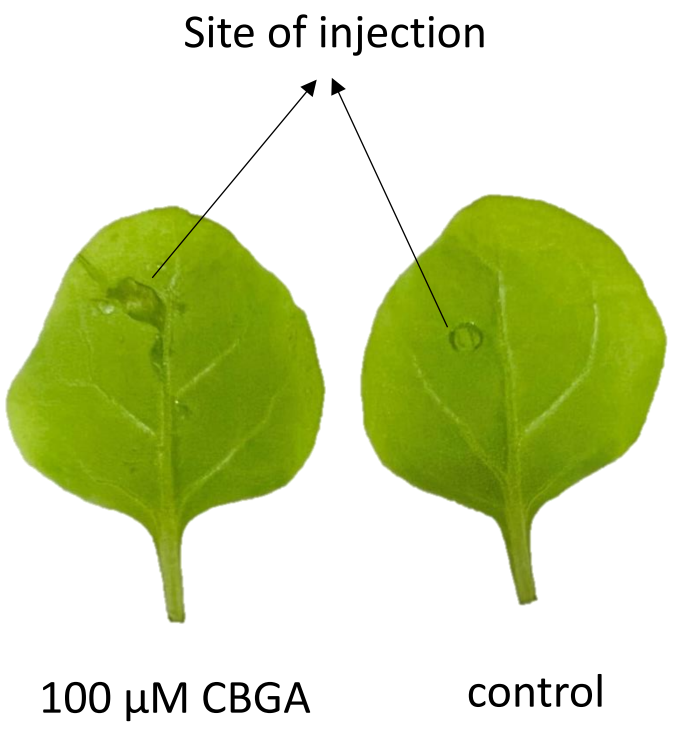

Figure S4. *N. benthamiana* leaf showing damage only at the site of injection of 100 µM of CBGA.

Table S1. List of primers used in this study.

Name	Sequence (5’ to 3’)	Purpose
CsOLS_F	CGTACCTAGGATGAATCATCTTCGTGCTGAGG	Cloning
CsOLS_R	ATCGCATGCTTAATATTTGATGGGAACACTACGC	Cloning
TNos_F	CGTAGGATCCGAGCTCGAATTTCCCCGAT	Cloning
TNos_R	ATCAGATCTCGAATTCCCGATCTAGTAACATAGATG	Cloning
pAtRBCS2B_F	CGTACTCGAGCTTTACCCTAACTACTCCTTTC	Cloning
pAtRBCS2B_R	ATCCCTGCAGGACTTCTTCTTGTTGTTTCTCTTCTTC	Cloning
CsAAE1_F	CGTAGTCGACATGGGTAAGAATTACAAGTCCCTG	Cloning
CsAAE1_R	ATCCATATGTCATTCAAAGTGAGAAAATTGCTGGCGC	Cloning
CsOAC_F	CACCATGGCAGTGAAGCATTTGATTG	Cloning
CsOAC_R	CTACTTTCGTGGTGTGTAGTCAAA	Cloning
Cs_CsPT1_F	CACCATGGGACTCTCATCAGTTTG	Cloning
Cs_CsPT1_R	TTATATGAAAACATATACTAAATATTCAGCATAATAAAGC	Cloning
Cs_CsPT4_F	CACCATGGGACTCTCATTAGTTTG	Cloning
Cs_CsPT4_R	TTATATAAATACATATACAAAGTATTCAGCA	Cloning
q_CsOLS_F	CCGGCCTCCGTTCTCGCCA	qPCR
q_CsOLS_R	GAGTTTGCATCTCGTGCTCCACCAATC	qPCR
q_CsAAE_F	GCCCTAGGTATCACCTCTGAAGTTGCTGAGA	qPCR
q_CsAAE_R	GGTATCCAAGCAGGAGGGGCAGG	qPCR
q_Actin_F	AATGGTCAAGGCTGGGTTTG	qPCR and Semi-quantitative PCR

q_Actin_R	TCCATGTCATCCCAGTTGCT	qPCR and Semi-quantitative PCR
Sq_CsOLS_F	AGTTGGTGTCAACTGGGCAA	Semi-quantitative PCR
Sq_CsOLS_R	CACTACGCACGACCACTCTT	Semi-quantitative PCR
Sq_CsAAE_F	GTAGACTGGCCGAGATCGTG	Semi-quantitative PCR
Sq_CsAAE_R	GGAAGCCATTCACTACCCCC	Semi-quantitative PCR

Table S2. Details of product ions and MS parameters.

**Product**	**Ion used for PRM**
Olivetol	181
Olivetolic acid	223
Divarinol	151
Divarinic acid	195
2,4-Dihydroxy-6-hexylbenzoic acid	237
Homoorsellinic acid	181
2,4-Dihydroxy-6-butylbenzoic acid	209
Cannabigerolic acid	359
**PRM parameters**	
Resolution	35000
Maximum IT	130 ms
Isolation window	1.0 m/z
(N)CE	10

Table S3. List of substrates used for infiltration and their respective products formed.

Chain Length	IUPAC ID of substrates	Formula	Product formed	Molecular weight of the product (g/mol)	Signature mass spec ions of the product
C3	Propanoic acid	$C_3H_6O_2$	2,4-Dihydroxy-6-ethylbenzoic acid (Homoorsellinic acid)	182	93, 95, 137, 181
C4	Butanoic acid	$C_4H_8O_2$	2,4-Dihydroxy-6-propylbenzoic acid (Divarinic acid)	196	107, 109, 151, 195
C5	Pentanoic acid	$C_5H_{10}O_2$	2,4-Dihydroxy-6-butylbenzoic acid	210	121, 123, 165, 209
C6	Hexanoic acid	$C_6H_{12}O_2$	2,4-Dihydroxy-6-pentylbenzoic acid (Olivetolic acid)	224	135, 137, 179, 223
C7	Heptanoic acid	$C_7H_{14}O_2$	2,4-Dihydroxy-6-hexylbenzoic acid	238	149, 151, 193, 237
